# One-pot Synthesis of 6-Aza-chromone Derivatives Through Cascade Carbonylation-Sonogashira-Cyclization

**DOI:** 10.1038/s41598-017-04693-7

**Published:** 2017-06-30

**Authors:** Gang Cheng, Yingbei Qi, Xiaoqian Zhou, Rong Sheng, Yong-Zhou Hu, Youhong Hu

**Affiliations:** 1 College of Pharmaceutical Sciences, Zhejiang University, Hangzhou, 310058 China; 2 State Key Laboratory of Drug Research, Shanghai Institute of Materia Medica, Chinese Academy of Sciences, 555 Zu Chong Zhi Road, Shanghai, 201203 China

## Abstract

We developed an efficient synthesis of aza-chromones from 3-iodo-4-(1*H*)-pyridones and terminal acetylenes via a cascade carbonylation-Sonogashira-cyclization reaction. By controlling the use of bases, both 6-aza-chromones **5** and 3-(4-oxo-1,4-dihydroquinoline-3-carbonyl)-4*H*-pyrano[3,2-*c*]quinolin-4-ones **6** could be selectively obtained in moderate to good yields.

## Introduction

**Figure 1:**
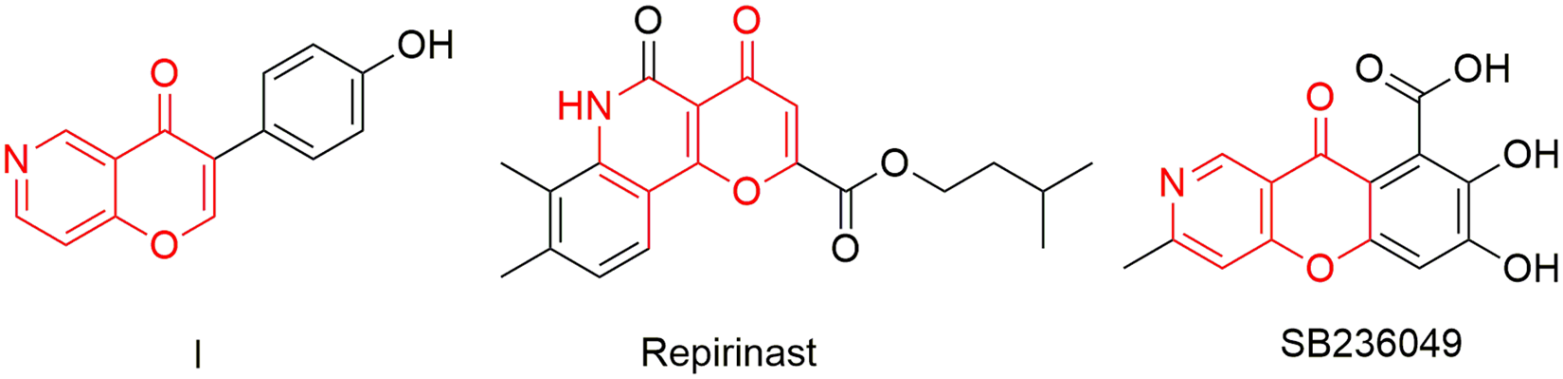
Selected examples of biological active and naturally occurring compounds containing azachromone units.

**Figure 2:**
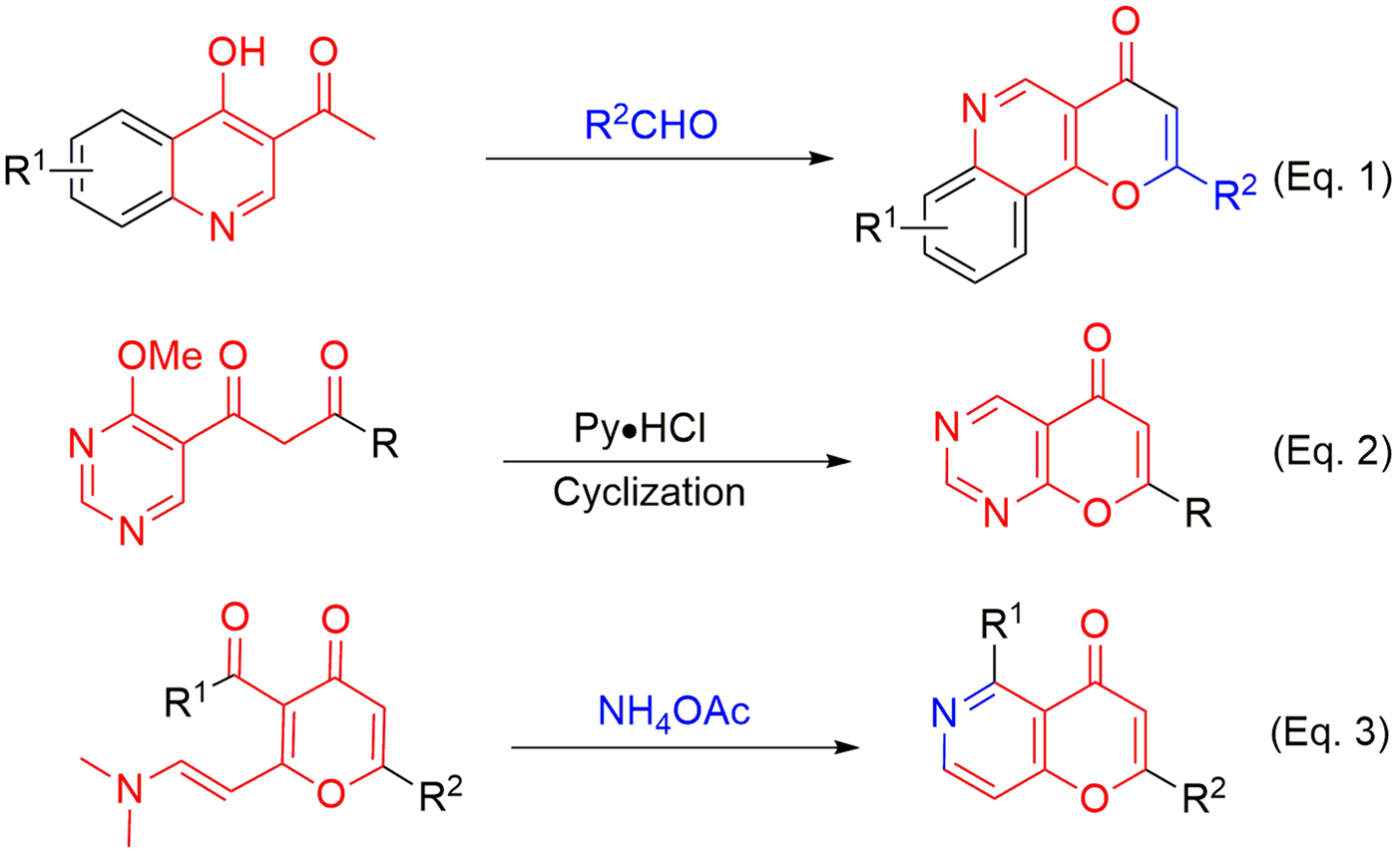
Reported methods for preparation of 6-aza-chromone.

Chromone and chromone derivatives are ubiquitous structures that constitute a variety of naturally occurring and synthetic bioactive compounds^1^. Among them, 6-aza-chromones are a class of interesting compounds that display various biological activities. For example (Fig. 1), compound **I** can inhibit bromodomain and extra terminal domain (BET) proteins with the potential as cancer therapeutic agents^2^; repirinast is an antiallergic drug used for bronchial asthma^3^; and SB236049 is a metallo-β-lactamase inhibitor that can overcome bacteria resistance to beta-lactam antibiotics^4, 5^. However, to date only few synthetic routes to this interesting scaffold been reported. These include the condensation of 3-acetyl-quinolinone with benzaldehyde (Fig. 2 and Eq. 1)^6, 7^, the cyclization of 1-(*ortho*-hydroxyaryl)-1,3-diketone (Fig. 2 and Eq. 2)^8^, and the condensation of 3-carbonyl-2-(2-(dimethylamino)vinyl)-4*H*-pyran-4-one with ammonium acetate (Fig. 2 and Eq. 3)^9^. The limited number of methodologies restricts the diversification and development of these compounds. In the context of our ongoing efforts to develop new methods for generating these diversified natural-product-like scaffolds^10–14^, we report herein a new facile synthetic method for 6-azachromone derivatives from 3-iodo-4-(1*H*)-pyridones and terminal acetylenes via cascade CO insertion, Sonogashira coupling and cyclization.

**Figure 3:**
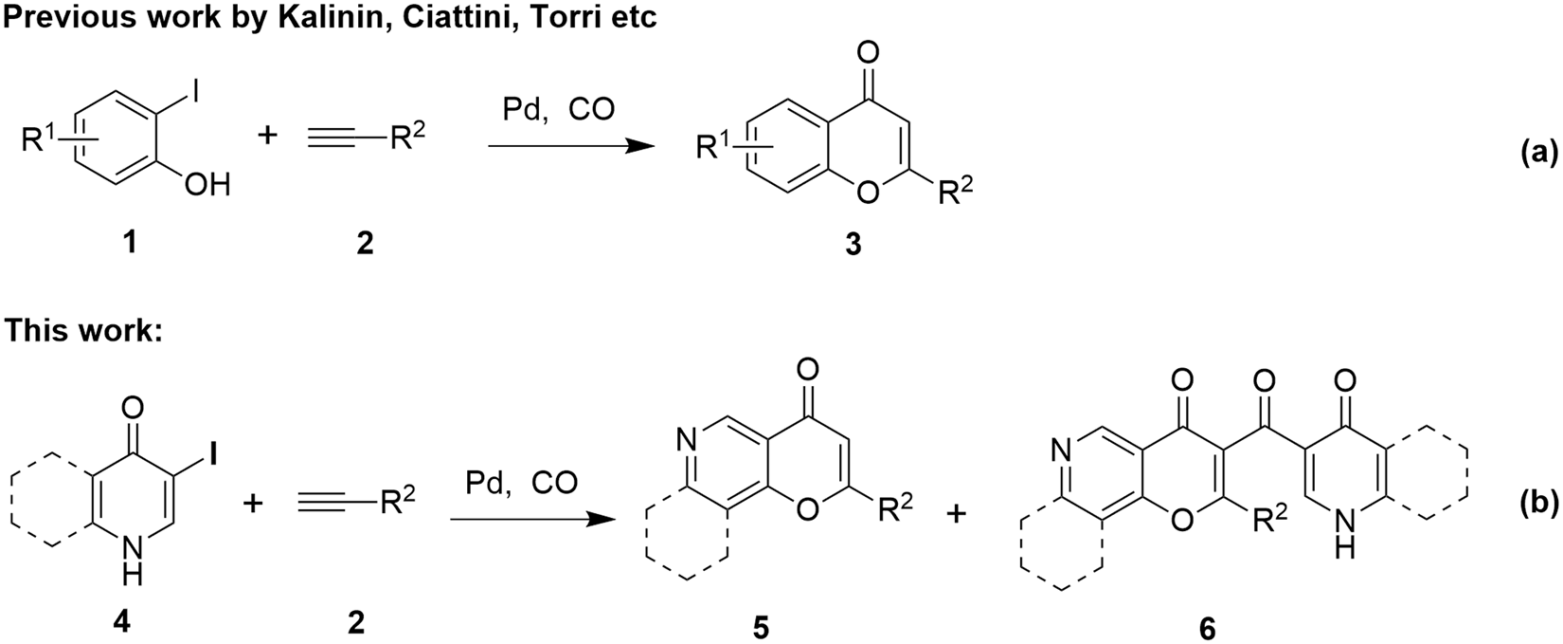
Cascade carbonylative Sonogashira-cyclization of o-iodophenol or 3-iodo-4-(1*H*)-pyridone.

Recently, palladium catalyzed carbonylative Sonogashira cross-couplings have attracted much attention^15–20^, and have been successfully applied in the synthesis of chromones using *o*-iodophenols and terminal acetylenes^21–27^. We have envisioned that a similar strategy could be applied to 3-iodo-4-(1*H*)-pyridone substrates to construct diverse 6-aza-chromone derivatives (**5**) (Fig. 3).

## Results and Discussion

**Figure 4:**
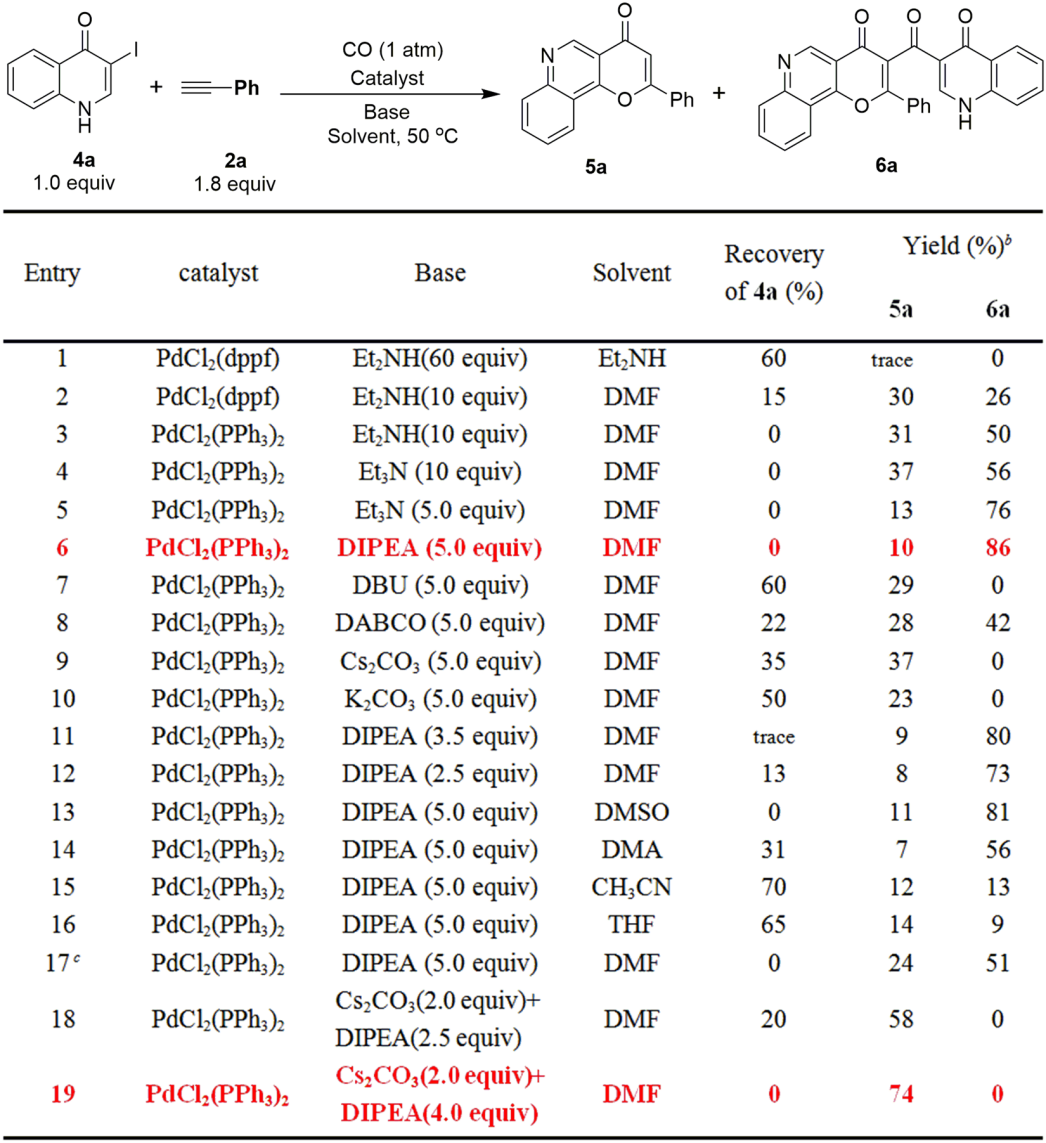
Optimization of the reaction conditions for the selective synthesis of **5a** or **6a**. ^*a*^Reaction condition (unless otherwise noted): **4a** (0.2 mmol), **2a** (0.36 mmol), catalyst (5 mol%), Solvent (1 mL), base, CO (1 atm), stirred at 50 °C for about 14 h. ^*b*^isolated yield. ^*c*^Using 1 atm N_2_ and Mo(CO)_6_ instead of 1 atm CO.

Our investigation started with the reaction of 3-iodo-quinolinone (**4a**) and phenylacetylene (**2a**). Initially, **4a** and **2a** were treated with Et_2_NH (as base and solvent)^21^ and PdCl_2_(dppf) (5 mol%) under 1 atm CO at 50 °C, but only a trace amount of product **5a** was observed, presumably due to the poor solubility of **4a** in Et_2_NH (Fig. 4, entry 1). In order to improve the solubility, we ran the reaction in DMF with an excess of Et_2_NH (10 equiv) as base; under these conditions, product **5a** could be obtained in 30% isolated yield, together with another unexpected compound **6a** in 26% yield. (Fig. 4, entry 2, the structure of **6a** was verified by single crystal X-ray diffraction, see Supplementary Information, Fig. 1S). Replacement of PdCl_2_(dppf) with PdCl_2_(PPh_3_)_2_ led to, allowed full conversion of **4a** and the yields of **5a** and **6a** were improved to 31% and 50%, respectively (Fig. 4, entry 3). Subsequently, different bases, including Et_3_N, DIPEA, DBU, DABCO, Cs_2_CO_3_ and K_2_CO_3_ were screened for this reaction. Using 5 equiv of Et_3_N or DIPEA as base, **4a** can be completely consumed and give **6a** in good yields (76% and 86%, respectively, Fig. 4, entries 5, 6). However, when DBU, DABCO, Cs_2_CO_3_ or K_2_CO_3_ was used, **4a** reacted partially react and produced **5a** and **6a** in low to moderate yields (23–42%, Fig. 4, entries 7–10). Reduction of the amount of DIPEA decreased the yield of **6a** (Fig. 4, entries 11, 12). Next, the effect of different solvents (DMSO, DMA, CH_3_CN, THF) was studied. Replacement of DMF with DMSO or DMA only slightly decreased the yield of **6a** (Fig. 4, entries 13, 14), while using CH_3_CN or THF as the solvent greatly lower the yield for both **5a** and **6a** (Fig. 4, entries 15, 16), probably due to the poor solubility of substrate **4a** in these solvents. Further replacement of CO gas (1 atm) with CO donor Mo(CO)_6_ gave inferior results (Fig. 4, entry 17). In summary, the optimized conditions (Condition **B**, Fig. 4, entry 6, shown in red) for the selective preparation of **6a** are PdCl_2_(PPh_3_)_2_ (5 mol%), DIPEA (5 equiv) in DMF under CO (1 atm) at 50 °C.

Following optimization of reaction conditions for the preparation of **6a**, we began searching for the optimal conditions for the preparation of **5**. It could be noted that when excessive amount of Cs_2_CO_3_ was used as a base only **5a** was selectively obtained in 37% yield, albeit 35% of **4a** was recovered (Fig. 4, entry 9). Our previous result indicated that DIPEA can promote the full conversion of **4a**, therefore we envisioned that the addition of DIPEA and Cs_2_CO_3_ together might achieve better yield of **5a**. Indeed, when DIPEA was used together with Cs_2_CO_3_ both the yield for **5a** (58%) and the conversion rate of **4a** were improved (Fig. 4, entry 18). When 4 equiv of DIPEA and 2 equiv of Cs_2_CO_3_ were used as bases, **4a** was completely consumed and **5a** was obtained in a yield of 74%. (Fig. 4, entry 19, condition **A**, shown in red).

**Figure 5:**
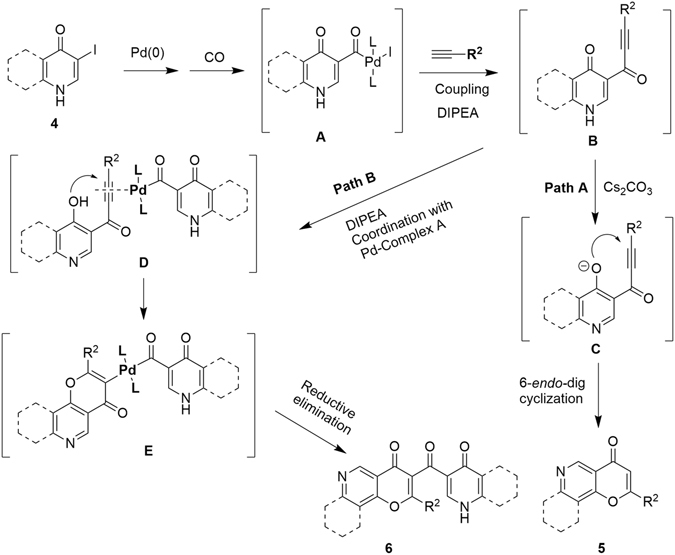
Plausible mechanism for the generation of product **5** and **6**.

Based on the above results, a reaction mechanism has been proposed (Fig. 5). Iodoquinoline substrate **4** first undergoes a consecutive oxidative addition and CO insertion to give Pd(II) complex **A**. Sonogashira coupling of **A** with terminal alkyne can generate intermediate propynone **B**. Deprotonation of **B** and the subsequent rearrangement can provide **C**, which undergoes 6-*endo*-dig cyclization to give product **5** (**Path A**). Intermediate **B** can also coordinate with another molecule of Pd(II) complex **A** to form Pd(II) complex **D**; Cyclization of **D** can afford intermediate **E**, which is followed by reductive elimination **to** give product **6** (**Path B**).

An interesting phenomenon about this reaction is that the use of different base leads to the production of compound **5** or **6** selectively, we speculate that DIEPA might facilitate a carbonylative Sonogashira coupling process to generate intermediate **B**, but have less effect on the deprotonation of **B** to **C**. While Cs_2_CO_3_ is not an optimum base for the carbonylative Sonogashira coupling, it is a stronger base, which promotes the deprotonation of **B** to generate phenoxide anion intermediate **C**, thus favors the self-cyclization to afford product **5**.

**Figure 6:**
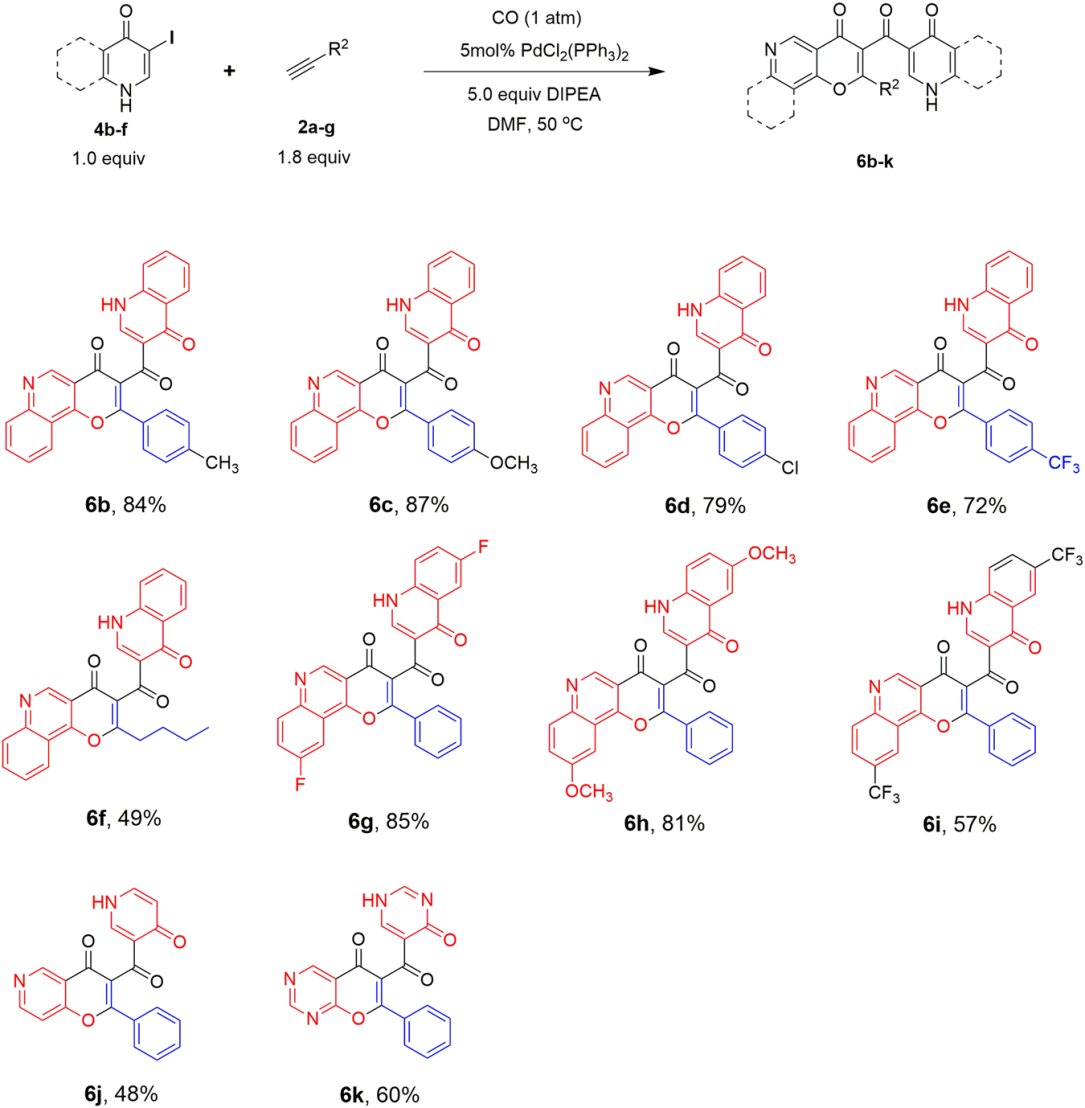
Synthesis of compounds **6b–k**. ^*a*^Reaction conditions: **4** (0.2 mmol), **2** (1.8 equiv), PdCl_2_(PPh_3_)_2_ (5 mol %), 5.0 equiv DIPEA, DMF (1 mL), CO (1 atm), stirred at 50 °C. ^*b*^Isolated yield.

With the optimized reaction conditions for path **B** in hand, the substrate scope of alkynes **2** was investigated. As shown in Fig. 6, both electron-donating and electron-withdrawing groups and substituted phenyl acetylenes can afford the desired products in good yields (72–87%, **6b**–**6e**), while hexyne only gave **6f** in moderate yield (49%), indicating the aromatic alkynes are more favorable substrates than aliphatic alkynes for this reaction. Subsequently, phenylacetylene **2a** was reacted with different substituted iodoquinolines (**4g–4i**) to explore the effect of substituents on the iodoquinoline ring. These reactions proceed smoothly with moderate to good yields (57%–85%), indicating the good tolerance of different substituents on iodoquinoline. In addition, using **4j** or **4k** as substrate also produces the corresponding product **6j** or **6k** with the yields of 48% and 60%, respectively, indicating the wide substrate scope of this reaction.

**Figure 7:**
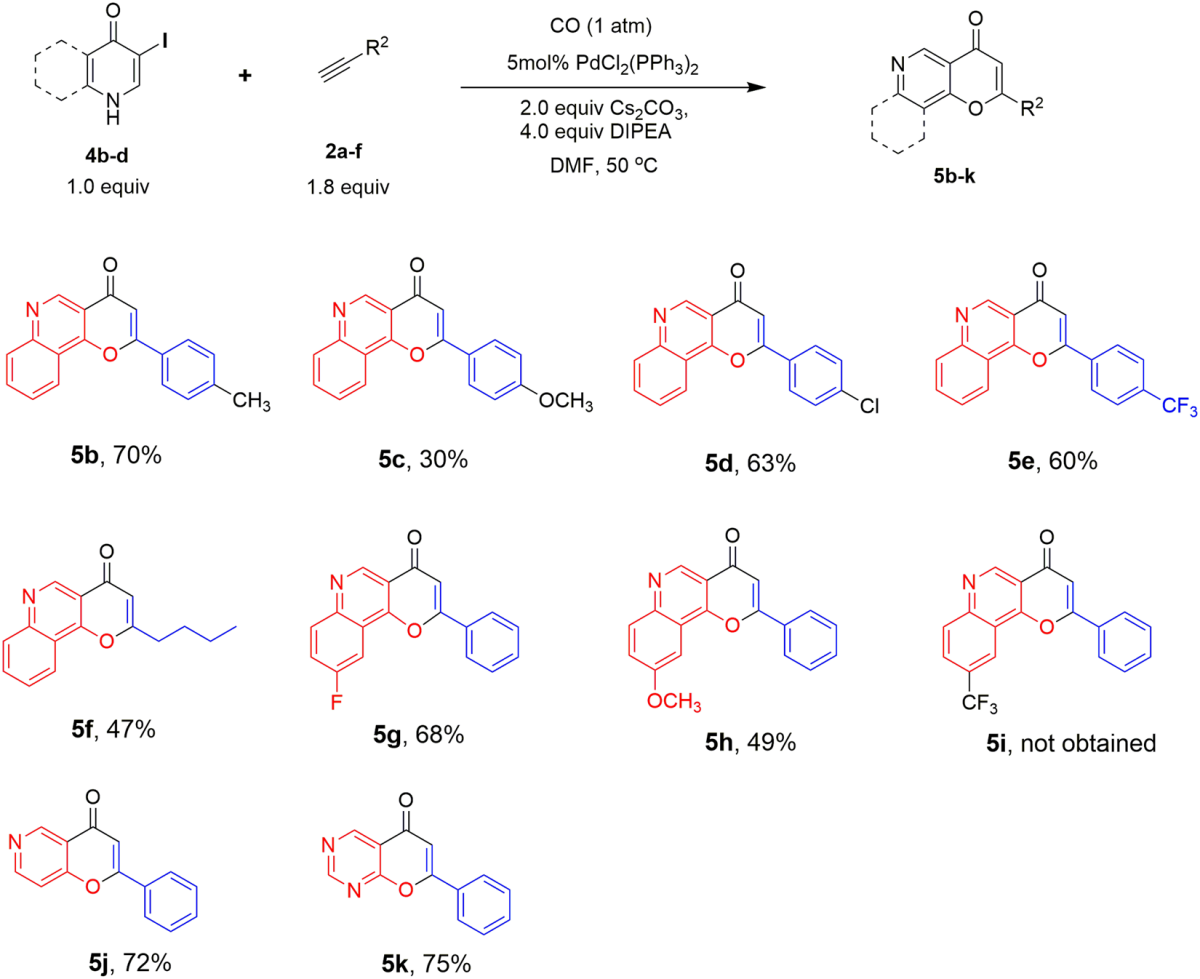
Synthesis of compounds **5b–5k**. ^*a*^Reaction condition: **4** (0.2 mmol), **2** (1.8 equiv), PdCl_2_(PPh_3_)_2_(5 mol %), 2.0 equiv Cs_2_CO_3_, 4.0 equiv DIPEA, DMF (1 mL), CO (1 atm), stirred at 50 °C. ^*b*^Isolated yield.

In a similar manner, we applied the optimized reaction conditions for path **A** to synthesize compound **5b–5k** (Fig. 7). All the reactions proceeded to give the corresponding products in moderate to good yields (30%–75%) except compound **5i**. The inferior yield of **5c** (30%) in comparison with **5d** (63%) suggests that an electron donating group on phenylacetylene is unfavourable for the production of compound **5**. Failure of the synthesis of compound **5i is** likely due to the strong electron withdrawing effect of CF_3_, which decreases the stability of **5i**. Mono heterocycle substrates 3-iodo-4-(1*H*)-pyridone and 5-iodopyrimidin-4(1 *H*)-one also gave corresponding products **5j** and **5k** in good yields (72%, 75%), indicating the good tolerance of various substrates in this transformation.

## Conclusions

In summary, we have developed an efficient method for the synthesis of 6-aza-chromone derivatives through cascade Carbonylation-Sonogashira-Cyclization reactions. Using different bases, both 6-aza-chromone derivatives **5** and **6** can be synthesized selectively in good yields. Further applications of these methods and the biological activities of these compounds are under investigation.

## Experimental Section

### General procedure A (Condition A): Synthesis of 5

A test tube equipped with a magnetic stir bar and fitted with a septum, was charged with 3-iodo substrates (0.2 mmol), Cs_2_CO_3_ (2.0 equiv), DIPEA (4.0 equiv), PdCl_2_(PPh_3_)_2_ (5 mol%). The test tube was evacuated and backfilled with CO (repeated 3 times) and then the alkyne (0.36 mmol) was added via syringe. The reaction mixture was heated to 50 °C until the starting material was completely consumed as monitored by TLC (typically 14 h). The reaction mixture was then cooled to room temperature, diluted with ethyl acetate, washed with water, concentrated under reduced pressure and purified by column chromatography (silica gel) to afford the corresponding compound **5**.

### General procedure B (Condition B): Synthesis of 6

A test tube equipped with a magnetic stir bar and fitted with a septum, was charged with 3-iodo substrates (0.2 mmol), DIPEA (5.0 equiv), PdCl_2_(PPh_3_)_2_ (5 mol%). The test tube was evacuated and backfilled with CO (repeated 3 times) and then the alkyne (0.36 mmol) was added via syringe. The reaction mixture was heated to 50 °C until the starting material was completely consumed as monitored by TLC (typically 14 h). The reaction mixture was then cooled down to room temperature, diluted with ethyl acetate, washed with water, concentrated under reduced pressure and purified by column chromatography (silica gel) to afford the corresponding compounds **6**.

## Electronic supplementary material

Supplementary Information
