# Supplementary material for: One-pot Synthesis of 6-Aza-chromone Derivatives Through Cascade Carbonylation-Sonogashira-Cyclization
Source: Sci Rep. 2017 Jun 30;7:4398. doi: 10.1038/s41598-017-04693-7 (PMC5493695; doi:10.1038/s41598-017-04693-7)
Supplement: Supplementary file 1 — Supplementary Information [file 41598_2017_4693_MOESM1_ESM.pdf]

One-pot Synthesis of 6-Aza-chromone Derivatives Through Cascade Carbonylation-Sonogashira-Cyclization

Gang Cheng,[1] Yingbei Qi,[1] Xiaoqian Zhou,[1] Rong Sheng,[1] Yong-Zhou Hu,[1] Youhong Hu[2]

[1] College of Pharmaceutical Sciences, Zhejiang University, Hangzhou 310058, China.

[2] State Key Laboratory of Drug Research, Shanghai Institute of Materia Medica, Chinese Academy of Sciences, 555 Zu Chong Zhi Road, Shanghai 201203, China. Correspondence and requests for materials should be addressed to R.S. (email: shengr@zju.edu.cn) or Y.Z.Hu. (e-mail: huyz@zju.edu.cn)

Table of contents

S1 Table of contents

S2 Figure 1S and Materials and General Procedures

S3 Synthesis of 6a-6k

S7 Synthesis of 5a-5k

S9 1H-NMR spectra and ^{13}C-NMR spectra

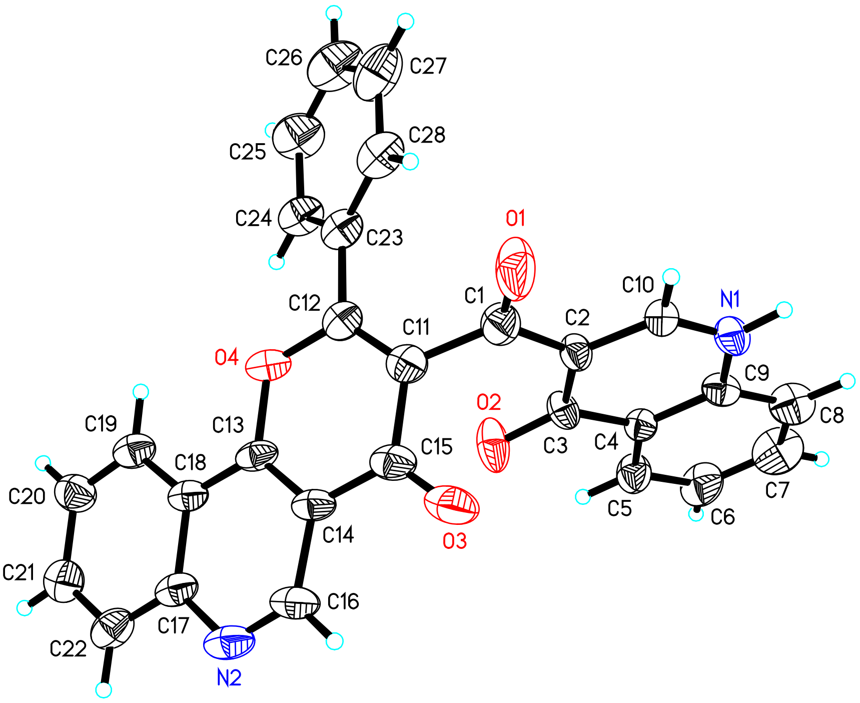

Figure 1S. Crystal structure of **6a**.Ellipsoid probability: 50%.

Materials and General Procedures

All the solvents and reagents were used directly as obtained commercially unless otherwise noted.^{1}H NMR (500 MHz) and ^{13}CNMR (100 MHz) spectra were determined in $CDCl_3$ unless otherwise specified. Chemical shifts (δ) are expressed in ppm relative to TMS as internal standard. The HRMS of all final products were measured on an Agilent 1290 HPLC-6224 Time of Fight Mass Spectrometer.

General procedure A (Condition A): Synthesis of 5

A test tube equipped with a magnetic stir bar and fitted with a septum, was charged with 3-iodide substrates (0.2 mmol), Cs_2CO_3 (2.0 equiv), DIPEA (4.0 equiv), $PdCl_2(PPh_3)_2$(5mol%). The test tube was evacuated and backfilled with CO (repeated 3 times) and then the alkyne (0.36 mmol) was added via syringe. The reaction mixture was heated to 50°C until the starting material was completely consumed as monitored by TLC. The reaction mixture was then cooled to room temperature, diluted with ethyl acetate, washed with water, concentrated under reduced pressure and purified by column chromatograph (silica gel) to afford the corresponding compound **5**.

General procedure B (Condition B): Synthesis of 6

A test tube equipped with a magnetic stir bar and fitted with a septum, was charged with 3-iodoquinoline (0.2 mmol), DIPEA (5.0 equiv), $PdCl_2(PPh_3)_2$(5 mol%). The test tube was evacuated and backfilled with CO (repeated 3 times) and then the alkyne (0.36 mmol) was added via syringe. The reaction mixture was heated to 50°C until the starting material was completely consumed as monitored by TLC. The reaction mixture was then cooled down to room temperature, diluted with ethyl acetate, washed with water, concentrated under reduced pressure and purified by column chromatograph to afford the corresponding compounds **6**.

Synthesis of 6a-k

3-(4-oxo-1, 4-dihydroquinoline-3-carbonyl)-2-phenyl-4*H*-pyrano[3,2-*c*]quinolin-4-one (6a)
Using 3-iodoquinoline and phenylacetylene as substrates, followed the general procedure A and purified by flash chromatography (silica gel, 20:1 CH_2Cl_2/MeOH) to afford 38.3 mg of **6a** (86%) as a white solid. **^{1}H NMR** (DMSO-*d$_6$)* δ 12.77 (brs, 1H), 9.35 (s, 1H), 8.73 (s, 1H),8.56 (d, *J* = 7.5 Hz, 1H), 8.32 (s, 1H), 8.24 (d, *J* = 8.4 Hz, 1H), 8.10-8.02 (m, 2H), 7.89 (t, *J* = 7.2 Hz, 1H), 7.85-7.65 (m, 4H), 7.57-7.49 (m, 3H), 7.43 (t, *J* = 7.2 Hz, 1H). **^{1}H NMR** ($CDCl_3$ + CD_3OD): δ11.65 (brs, 1H), 9.68 (brs, 1H), 8.56-8.48 (m, 2H), 8.28 (d, *J* = 8.4 Hz, 1H), 7.97 (t, *J* = 7.2 Hz, 1H), 7.86-7.73 (m, 4H), 7.53-7.40 (m, 3H), 7.39-7.26 (m, 2H), 7.02 (t, *J* = 8.1 Hz, 1H). **^{13}C NMR** ($CDCl_3$+CD_3OD): δ189.86, 175.97, 175.73, 160.58, 158.96, 149.28, 147.88, 144.71, 138.83, 132.73, 132.59, 131.38, 131.25, 129.32, 128.96, 128.85, 128.60, 127.96, 127.81, 126.13, 125.47, 122.07, 118.42, 118.33, 114.28. **HRMS (ESI)**: m/z calcd for $(C_{28}H_{16}N_2O_4+H)^+$: 445.1188; found: 445.1190

3-(4-oxo-1,4-dihydroquinoline-3-carbonyl)-2-(p-tolyl)-4*H*-pyrano[3,2-*c*]quinolin-4-one (6b)
Using 3-iodoquinoline and 4-methylphenylacetylene as substrates, followed the general procedure A and purified by flash chromatography (silica gel, 20:1 CH_2Cl_2/MeOH) to afford 38.5 mg of **6b** (86%) as a white solid. **^{1}H NMR** (DMSO-*d$_6$*) (500 M): δ 12.82 (brs, 1H), 9.35 (s, 1H), 8.73 (s, 1H), 8.56 (d, *J* = 7.5 Hz, 1H), 8.24 (d, *J* = 8.5 Hz, 1H), 8.08-8.05 (m, 2H), 7.90 (t, *J* = 7.5 Hz, 1H), 7.77-7.66 (m, 4H), 7.42 (t, *J* = 8.0Hz, 1H), 7.33 (t, *J* = 8.0 Hz, 1H), 2.35 (s, 3H). **^{13}C NMR** ($CDCl_3$+CD_3OD) δ190.03, 175.99, 175.57, 160.72, 158.81, 149.06, 147.73, 144.73, 141.92, 138.83, 132.73, 132.51, 129.32, 128.70, 128.40, 128.34, 127.90, 127.73, 126.04, 125.42, 121.99, 118.38, 118.34, 118.18, 114.14, 21.08; **HRMS (ESI):** m/z calcd for $(C_{29}H_{18}N_2O_4+H)^+$: 459.1345; found: 459.1349

2-(4-methoxyphenyl)-3-(4-oxo-1,4-dihydroquinoline-3-carbonyl)-4*H*-pyrano[3,2-*c*]quinolin-4-one (6c).
Using 3-iodoquinoline and 4-methoxyphenylacetylene as substrates, followed the general procedure A and purified by flash chromatography (silica gel, 20:1 CH_2Cl_2/MeOH) to afford 41.3 mg of **6c** (87%) as a white solid. **^{1}H NMR** (DMSO-*d$_6$*) (500 M) δ 12.80 (brs, 1H), 9.34 (s, 1H), 8.74 (s, 1H), 8.58 (d, *J* = 7.5 Hz, 1H), 8.23 (d, *J* = 8.0 Hz, 1H), 8.08-8.04 (m, 2H), 7.90 (t, *J* = 8.0 Hz, 1H), 7.79-7.7.74 (m, 3H),

7.69 (d, J = 8.0 Hz, 1H), 7.43 (t, J = 8.0 Hz, 1H), 7.08 (d, J = 6.5 Hz, 1H), 3.80 (s, 3H). **^{13}C NMR** ($CDCl_3$+ CD_3OD) δ 190.18, 175.88, 175.83, 162.04, 160.65, 158.74, 149.22, 147.80, 144.91, 138.63, 132.66, 132.52, 130.37, 128.97, 127.92, 127.61, 125.92, 125.42, 123.38, 122.02, 118.47, 118.31, 118.14, 114.22, 114.16, 55.18; **HRMS (ESI):** m/z calcd for $(C_{29}H_{18}N_2O_5+H)^+$: 475.1294; found: 475.1297.

2-(4-chlorophenyl)-3-(4-oxo-1,4-dihydroquinoline-3-carbonyl)-4*H*-pyrano[3,2-*c*]quinolin-4-one (6d).

Using 3-iodoquinoline and 4-chlorophenylacetylene as substrates, followed the general procedure A and purified by flash chromatography (silica gel, 20:1 CH_2Cl_2/MeOH) to afford 37.8 mg of **6c** (79%) as a solid. **^{1}H NMR** (DMSO-d_6)δ: 12.86 (brs, 1H), 9.36 (s, 1H), 8.73 (s, 1H), 8.57 (d, J = 8.5 Hz, 1H), 8.24 (d, J = 8.5 Hz, 1H), 8.09-8.05 (m, 2H),7.90 (t, J = 8.0 Hz, 1H), 7.82 (d, J = 8.0 Hz, 2H), 7.75 (t, J = 8.0 Hz, 1H), 7.69 (d, J = 8.0 Hz, 1H), 7.62 (d, J = 8.0 Hz, 2H), 7.44 (t, J = 8.0 Hz, 1H); **^{13}C NMR** (DMSO-d_6) (125 M) δ: 189.15, 175.21, 175.09, 158.63, 157.90, 149.91, 147.76, 145.79, 139.82, 136.73, 133.43, 133.33, 130.79, 130.73, 130.21 129.93, 129.67, 128.83, 128.15, 126.14, 125.85, 122.84, 119.91, 118.37, 114.38; **HRMS (ESI):** m/z calcd for $(C_{28}H_{15}ClN_2O_4+H)^+$: 479.0799; found: 479.0808.

2-(4-trifluoromethylphenyl)-3-(4-oxo-1,4-dihydroquinoline-3-carbonyl)-4*H*-pyrano[3,2-*c*]quinolin -4-one (6e).

Using 3-iodoquinoline and 4-trifluoromethylphenylacetylene as substrates, followed the general procedure A and purified by flash chromatography (silica gel, 20:1 CH_2Cl_2/MeOH) to afford 36.9 mg of **6e** (72%) as a white solid. **^{1}H NMR** (DMSO-d_6+CD_3OD) (500 M) δ: 12.9 (brs, 1H), 9.37 (s, 1H), 8.74 (s, 1H), 8.56 (d, J = 8.0 Hz, 1H), 8.24 (d, J = 8.5 Hz, 1H), 8.10-8.04 (m, 2H), 8.01 (d, J = 8.0 Hz, 2H), 7.94-7.86 (m, 3H), 7.75 (t, J = 7.0 Hz, 1H), 7.69 (d, J = 8.5 Hz, 1H), 7.43 (d, J = 8.5 Hz, 1H); **^{13}C NMR** (DMSO-d_6) (125 M) δ: 188.89, 175.30, 175.08, 158.71, 157.41, 149.94, 147.75, 145.91, 140.00, 135.85, 133.39, 131.50 (q, $^2J_{C\text{-}F}$ = 32.0 Hz), 131.05, 129.92, 128.84,128.17, 126.46, 126.12, 125.82, 125.30, 123.13, 122.86, 120.00, 118.34, 114.42; **HRMS (ESI):** m/z calcd for $(C_{29}H_{15}F_3N_2O_4+H)^+$: 513.1062; found: 513.1057.

2-butyl-3-(4-oxo-1,4-dihydroquinoline-3-carbonyl)-4*H*-pyrano[3,2-*c*]quinolin-4-one (6f).

Using 3-iodoquinoline and hexyne as substrates, followed the general procedure A and purified by flash chromatography (silica gel, 25:1 CH_2Cl_2/MeOH) to afford 20.8 mg of **6f** (72%) as a solid. **^{1}H NMR** ($CDCl_3$)δ:11.86 (brs, 1H), 9.62 (s, 1H), 8.58 (s, 1H), 8.43 (d, J = 7.0 Hz, 1H), 8.23 (d, J = 8.0 Hz, 1H), 7.94 (t, J = 7.5 Hz, 1H), 7.82 (d, J = 8.0 Hz, 1H), 7.76 (t, J = 7.5 Hz, 1H), 7.32 (s, 2H), 7.00 (s, 1H), 2.84 (t, J = 7.5 Hz, 2H), 1.86 (m, 2H), 1.45 (m, 2H), 0.92 (t, J = 7.5 Hz,3H); **^{13}C NMR** ($CDCl_3$) δ 189.33, 176.61, 175.62, 167.25, 159.01, 149.84, 148.07, 145.12, 138.40, 132.59, 129.77, 129.27, 128.00, 127.49, 125.78, 125.48, 121.95, 118.88, 118.35, 118.05, 114.43, 32.30, 29.71, 29.47, 22.42; **HRMS (ESI):** m/z calcd for $(C_{26}H_{20}ClN_2O_4+H)^+$: 425.1501; found: 425.1507.

9-fluoro-3-(6-fluoro-4-oxo-1,

4-dihydroquinoline-3-carbonyl)-2-phenyl-4*H*-pyrano[3,2-*c*]quinolin-4-one (6g).

Using 6-fluoro-3-iodoquinoline and phenylacetylene as substrates, followed the general procedure A and purified by flash chromatography (silica gel, 20:1 CH_2Cl_2/MeOH) to afford 40.8 mg of **6g** (85%) as a solid. **^{1}H NMR** (DMSO-d_6) δ 12.97 (s, 1H), 9.34 (s, 1H), 8.76 (s, 1H), 8.32 (dd, *J* = 9.0, 5.0 Hz, 1H), 8.27 (dd, *J* = 8.5, 2.5 Hz, 1H), 7.98 (td, *J* = 8.5, 3.0 Hz, 1H), 7.82 (d, *J* = 8.0 Hz, 1H), 7.77 (dd, *J* = 9.0, 4.5 Hz, 1H), 7.75 (dd, *J* = 9.0, 3.0 Hz, 1H), 7.67 (td, *J* = 8.0, 2.5 Hz, 1H),7.60-7.50 (m, 3H); **^{13}C NMR** ($CDCl_3$+CD_3OD) δ: 189.46, 175.32, 175.05, 161.22 (d, $^1J_{C\text{-}F}$ = 250 Hz), 159.99 (d, $^1J_{C\text{-}F}$ = 248 Hz), 158.36 (d, $^4J_{C\text{-}F}$ = 4.9 Hz),147.03, 146.09, 144.51, 135.38, 131.00 (d, $^3J_{C\text{-}F}$ = 9.1 Hz),131.30, 130.96,129.40 (d, $^3J_{C\text{-}F}$ = 7.2 Hz), 129.10, 128.60, 128.42, 122.26 (d, $^2J_{C\text{-}F}$ = 25 Hz), 121.25 (d, $^2J_{C\text{-}F}$ = 25 Hz), 120.74 (d, $^3J_{C\text{-}F}$ = 8.0 Hz), 119.39 (d, $^3J_{C\text{-}F}$ = 9.9 Hz), 117.36, 114.44, 110.93 (d, $^2J_{C\text{-}F}$ = 23 Hz), 106.24 (d, $^3J_{C\text{-}F}$ = 24 Hz); **HRMS (ESI):** m/z calcd for $(C_{28}H_{14}F_2N_2O_4+H)^+$: 481.1000; found: 481.1007.

9-methoxy-3-(6-methoxy-4-oxo-1,4-dihydroquinoline-3-carbonyl)-2-phenyl-4*H*-pyrano[3,2-*c*]quinolin-4-one (6h).

Using 6-methoxy-3-iodoquinolineand phenylacetylene as substrates, followed the general procedure A and purified by flash chromatography (silica gel, 20:1 CH_2Cl_2/MeOH) to afford 40.8 mg of **6h** (81%) as a solid. **^{1}H NMR** (DMSO-d_6)δ 12.83 (brs, 1H), 9.21 (s, 1H), 8.67 (s, 1H), 8.16 (d, *J* = 9.0 Hz, 1H), 7.81 (d, *J* = 7.5 Hz, 2H), 7.75 (d, *J* = 3.0 Hz, 1H), 7.70 (dd, *J* = 9.0, 3.0 Hz, 1H), 7.64 (d, *J* = 9.0Hz, 1H), 7.58-7.49 (m, 4H), 7.36 (dd, *J* = 9.0, 3.0 Hz, 1H), 4.02 (s, 3H), 3.79 (s, 3H); **^{13}C NMR** ($CDCl_3$+CD_3OD) δ 190.22, 175.70, 175.50, 160.89, 159.53, 158.81, 157.86, 157.54, 144.87, 144.77, 143.94, 133.11, 131.35, 131.16, 130.17, 128.72, 128.39, 124.30, 123.29, 120.14, 119.23, 116.79, 114.25, 105.34, 100.19, 55.53, 55.22; **HRMS (ESI):** m/z calcd for $(C_{30}H_{20}N_2O_6+H)^+$: 505.1400; found: 505.1405.

3-(4-oxo-6-(trifluoromethyl)-1,4-dihydroquinoline-3-carbonyl)-2-phenyl-9-(trifluoromethyl)-4*H*-pyrano[3,2-*c*]quinolin-4-one (6i).

Using 6-trifluoromethyl-3-iodoquinoline and phenylacetylene as substrates, followed the general procedure A and purified by flash chromatography (silica gel, 20:1 CH_2Cl_2/MeOH) to afford 33.0 mg of **6i** (57%) as a yellow solid. **^{1}H NMR** ($CDCl_3$ +CD_3OD) δ 9.52 (s, 1H), 8.65 (s, 1H), 8.54 (s, 1H), 8.43 (s, 1H), 8.25 (d, *J* = 9.0 Hz, 1H), 8.04 (d, *J* = 9.0Hz, 1H), 7.73 (d, *J* = 8.5 Hz, 1H), 7.66 (d, *J* = 7.0 Hz, 2H), 7.51 (d, *J* = 8.5 Hz, 1H), 7.45-7.35 (m, 3H); **^{13}C NMR** ($CDCl_3$ + CD_3OD) δ 189.14, 175.19, 175.03, 161.07, 158.97, 150.27, 150.18, 145.69, 140.90, 131.46, 130.79, 130.30, 129.64 (q, $^2J_{C\text{-}F}$ = 33 Hz), 129.15, 128.93, 128.73, 128.72 (q, $^2J_{C\text{-}F}$ = 32 Hz), 127.442, 122.25 (q, $^2J_{C\text{-}F}$ = 33 Hz), 124.61 (q, $^3J_{C\text{-}F}$ = 7.5 Hz), 124.10, 121.90 (q, $^3J_{C\text{-}F}$ = 7.6 Hz), 119.93 (q, $^4J_{C\text{-}F}$ = 3.9 Hz), 118.73, 117.92, 114.81; **HRMS (ESI):** m/z calcd for $(C_{30}H_{14}F_6N_2O_6+H)^+$: 581.0936; found: 581.0945.

3-(4-oxo-1,4-dihydropyridine-3-carbonyl)-2-phenyl-4*H*-pyrano[3,2-*c*]pyridin-4-one (6j)

Using 3-iodo-4-hydroxylpyridine and phenylacetylene as substrates, followed the general procedure A and purified by flash chromatography (silica gel, 20:1 CH_2Cl_2/MeOH) to afford 33.0 mg of **6j** (48%) as

a yellow solid. **^{1}H NMR** (CD_3OD) δ 9.28 (s, 1H), 8.82 (d, *J* = 4.5 Hz, 1H), 8.38 (s, 1H), 7.73-7.67 (m, 2H),7.65 (d, *J* = 7.5 Hz, 2H), 7.52 (t, *J* = 7.5 Hz, 1H), 7.45 (t, *J* = 7.5 Hz, 2H), 6.44 (d, *J* = 7.0 Hz, 1H); **^{13}C NMR** (DMSO-d_6) δ 190.24, 189.53, 176.20, 175.21, 160.96, 160.18, 154.26, 149.10, 143.58, 138.47, 131.87, 131.84, 129.30, 128.88, 125.28, 122.25, 118.72, 113.61; **HRMS (ESI)**: m/z calcd for $(C_{20}H_{12}N_2O_4+H)^+$: 345.0870; found: 345.0871.

6-(4-oxo-1,4-dihydropyrimidine-5-carbonyl)-7-phenyl-5*H*-pyrano[2,3-*d*]pyrimidin-5-one (6k)

Using 5-iodopyrimidin-4-ol and phenylacetylene as substrates, followed the general procedure A and purified by flash chromatography (silica gel, 20:1 CH_2Cl_2/MeOH) to afford 36 mg of **6j** (60%) as a yellow solid. **^{1}H NMR** (DMSO-d_6+CD_3OD) δ 9.48 (s, 1H), 9.40 (s, 1H), 8.57 (s, 1H), 8.47 (s, 1H), 7.64-7.51 (m, 5H); **^{13}C NMR** (DMSO-d_6+CD_3OD) δ 188.66, 175.60, 165.56, 162.31, 162.28, 159.78, 158.60, 155.30, 132.42, 161.10, 129.49, 129.04, 126.98, 124.04, 115.73; **HRMS (ESI):** m/z calcd for $(C_{18}H_{10}N_4O_4+H)^+$: 304.0974; found: 304.0796.

Synthesis of 5a-k

2-phenyl-4*H*-pyrano[3,2-*c*]quinolin-4-one (5a).

Using 3-iodoquinoline and phenylacetylene as substrates, followed the general procedure B and purified by flash chromatography (silica gel, 2:1 petroleum ether/ethyl acetate) to afford 20.7 mg of **5a** (75%) as a white solid. **^{1}H NMR** ($CDCl_3$): δ 9.50 (s, 1H), 8.44 (d, *J* = 8.5 Hz, 1H), 8.16 (d, *J* = 8.0 Hz, 1H), 7.97 (t, *J* = 7.5 Hz, 2H), 7.87 (t, *J* = 8.0 Hz, 1H), 7.71 (t, *J* = 7.5 Hz, 1H), 7.60-7.53 (m, 3H), 6.94 (s, 1H). **^{13}C NMR** ($CDCl_3$+CD_3OD) δ177.41, 163.80, 158.88, 149.59, 147.91, 132.66, 132.32, 130.74, 129.39, 129.35, 128.10, 126.32, 121.97, 118.54, 114.82, 110.25; **HRMS (ESI):** m/z calcd for $(C_{18}H_{11}NO_2+H)^+$: 274.0868; found: 274.0865.

2-(*p*-tolyl)-4*H*-pyrano[3,2-*c*]quinolin-4-one (5b).

Using 3-iodoquinoline and 4-methylphenylacetylene as substrates, followed the general procedure B and purified by flash chromatography (silica gel, 2:1 petroleum ether/ethyl acetate) to afford 20.1mg of **5b** (70%) as a white solid. **^{1}H NMR** ($CDCl_3$): δ 9.60 (s, 1H), 8.55 (d, *J* = 8.5 Hz, 1H), 8.24 (d, *J* = 8.5 Hz, 1H), 7.94 (t, *J* = 7.5 Hz, 1H), 7.91 (d, *J* = 8.0 Hz, 2H), 7.78 (t, *J* = 7.5 Hz, 1H), 7.40 (d, *J* = 8.0 Hz, 2H), 6.98 (s, 1H), 2.49 (s, 3H); **^{13}C NMR** ($CDCl_3$) δ177.13, 163.54 , 158.67, 150.01, 148.25, 142.96, 132.25, 130.08, 129.96, 128.23, 127.77, 126.22, 121.92, 118.65, 114.99, 109.90, 21.63; **HRMS (ESI)**: m/z calcd for $(C_{19}H_{13}NO_2+H)^+$: 288.1025; found: 288.1023.

2-(4-methoxyphenyl)-4*H*-pyrano[3,2-*c*]quinolin-4-one (5c).

Using 3-iodoquinoline and 4-methoxyphenylacetylene as substrates, followed the general procedure B and purified by flash chromatography (silica gel, 2:1 petroleum ether/ethyl acetate) to afford 9.1mg of **5c** (30%) as a white solid. **^{1}H NMR** ($CDCl_3$ + CD_3OD): δ 9.51 (s, 1H), 8.52 (d, *J* = 8.0 Hz, 1H), 8.19 (d, *J* = 8.5 Hz, 1H), 7.95 (d, *J* = 9.0 Hz, 2H), 7.91 (td, *J* = 7.0, 1.5 Hz, 1H), 7.76 (t, *J* = 7.5 Hz, 1H), 7.06 (d, *J* = 9.0 Hz, 2H), 6.91 (s, 1H), 3.90 (s, 3H). **^{13}C NMR** ($CDCl_3$+ CD_3OD) δ 177.39, 163.81, 162.95, 158.79, 149.72 (d, *J* = 7.5 Hz), 148.10, 132.45 (d, *J* = 8.7 Hz), 129.58 (d, *J* = 5.9 Hz), 128.20, 127.91

(d, J = 5.9 Hz), 123.10 (d, J = 8.4 Hz), 121.92 (d, J = 4.0 Hz), 118.65, 114.82, 108.80 (d, J = 5.1 Hz), 55.57 (d, J = 3.1 Hz); **HRMS (ESI):** m/z calcd for $(C_{19}H_{13}NO_3+H)^+$: 304.0974; found: 304.0790.

2-(4-chlorophenyl)-4*H*-pyrano[3,2-*c*]quinolin-4-one (5d).

Using 3-iodoquinoline and 4-chlorophenylacetylene as substrates, followed the general procedure B and purified by flash chromatography (silica gel, 2:1 petroleum ether/ethyl acetate) to afford 19.3 mg of **5d** (63%) as a solid. ^{1}**H NMR** ($CDCl_3$ + CD_3OD): δ 9.50 (s, 1H), 8.50 (d, J = 8.0 Hz, 1H), 8.19 (d, J = 8.0 Hz, 1H), 7.96-7.90 (m, 3H), 7.77 (t, J = 8.0 Hz, 1H), 7.50 (dJ = 8.5 Hz, 2H), 6.97 (s, 1H). ^{13}**C NMR** ($CDCl_3$+ CD_3OD) δ 177.13, 162.79, 158.90, 149.05, 147.45, 138.57, 132.83, 129.52, 128.97, 128.83, 128.22, 127.51, 121.83, 118.34, 114.62, 110.14; **HRMS (ESI):** m/z calcd for $(C_{18}H_{10}ClNO_2+H)^+$: 308.0478; found: 308.0479.

2-(4-trifluomethylphenyl)-4*H*-pyrano[3,2-*c*]quinolin-4-one (5e).

Using 3-iodoquinoline and 4-trifluoromethylphenylacetylene as substrates, followed the general procedure B and purified by flash chromatography (silica gel, 2:1 petroleum ether/ethyl acetate) to afford 20.5 mg of **5e** (60%) as a solid. ^{1}**H NMR** ($CDCl_3$+ CD_3OD) (500 M): δ 9.60 (s, 1H), 8.53 (d, J = 7.5 Hz, 1H), 8.27 (d, J = 8.5 Hz, 1H), 8.13 (d, J = 8.0 Hz, 2H), 7.96 (t, J = 8.0 Hz, 1H), 7.88 (d, J = 8.5 Hz, 2H), 7.80 (t, J = 8.0 Hz, 1H), 7.07 (s, 1H); ^{13}**C NMR** ($CDCl_3$+ CD_3OD) δ 176.87, 161.77, 158.76, 149.96, 147.98, 134.39, 133.61 (q, $^2J_{C\text{-}F}$ = 32.8 Hz), 132.67, 129.87, 128.12, 126.36, 123.49 (q, $^1J_{C\text{-}F}$ = 271.0 Hz), 121.79, 118.36, 114.89, 111.80; **HRMS (ESI)**: m/z calcd for $(C_{19}H_{10}NO_2+H)^+$: 342.0742; found: 304.0780.

2-butyl-4*H*-pyrano[3,2-*c*]quinolin-4-one (5f).

Using 3-iodoquinoline and hexyne as substrates, followed the general procedure B and purified by flash chromatography (silica gel, 2:1 petroleum ether/ethyl acetate) to afford 11.9mg of **5f** (47%) as a solid. ^{1}**H NMR** ($CDCl_3$) (500 M) δ 9.54 (s, 1H), 8.41 (d, J = 8.0 Hz, 1H), 8.21 (d, J = 8.5 Hz, 1H), 7.90 (t, J = 8.0 Hz, 1H), 7.72 (t, J = 8.0 Hz, 1H), 6.38 (s, 1H), 2.78 (t, J = 7.5 Hz, 2H), 1.83 (m, 2H), 1.51 (t, J = 7.5 Hz, 1H), 1.01 (t, J = 7.5 Hz, 1H); ^{13}**C NMR** ($CDCl_3$) δ177.16, 169.56, 159.02, 149.92, 149.38, 132.16, 129.91, 127.66, 121.84, 118.53, 114.79, 113.00, 33.86, 28.93, 22.14, 13.75; **HRMS** (ESI): m/z calcd for $(C_{16}H_{15}NO_2+H)^+$: 254.1181; found: 254.1180.

9-fluoro-2-phenyl-4*H*-pyrano[3,2-*c*]quinolin-4-one (5g).

Using6-fluoro-3-iodoquinoline and phenylacetylene as substrates, followed the general procedure B and purified by flash chromatography (silica gel, 2:1 petroleum ether/ethyl acetate) to afford 19.8mg of **5g** (68%) as a solid. ^{1}**H NMR** ($CDCl_3$) δ 9.47 (s, 1H), 8.21 (dd, J = 9.5, 5.0 Hz, 1H), 8.00 (dd, J = 8.5, 3.0 Hz, 1H), 7.99-7.96 (m, 2H), 7.67 (t, J = 8.0 Hz, 2H), 7.64-7.57 (m, 3H), 7.01 (m, 1H). ^{13}**C NMR** ($CDCl_3$) δ176.84, 163.50, 161.20 (d, $^1J_{C\text{-}F}$ = 250.0 Hz), 158.29, 147.58, 147.17, 132.74 (d, $^3J_{C\text{-}F}$ = 9.1 Hz), 132.25, 131.01, 129.43, 126.34, 122.03 (d, $^2J_{C\text{-}F}$ = 25 Hz), 119.57, 115.36, 110.85, 106.15 (d, $^2J_{C\text{-}F}$ = 23.8 Hz); **HRMS** (ESI): m/z calcd for $(C_{18}H_{10}FNO_2+H)^+$: 292.0774; found: 292.0775.

9-methoxy-2-phenyl-4*H*-pyrano[3,2-*c*]quinolin-4-one (5h).

Using 6-methoxy-3-iodoquinoline and phenylacetylene as substrates, followed the general procedure B and purified by flash chromatography (silica gel, 2:1 petroleum ether/ethyl acetate) to afford 14.8 mg of **5h** (49%) as a solid. **^{1}H NMR** ($CDCl_3$) (500 M): δ 9.43 (s, 1H), 8.13 (d, *J* = 8.0 Hz, 1H),8.00-7.95 (m, 2H), 7.71 (d, *J* = 2.5 Hz, 1H), 7.64-7.57 (m, 3H), 7.54 (dd, *J* = 8.0, 2.5 Hz, 1H), 6.98 (s, 1H), 4.05 (s, 3H);**^{13}C NMR** ($CDCl_3$) δ 177.24, 163.35, 158.82, 157.93, 145.84, 145.47, 132.09, 131.45, 131.31, 129.41, 126.30, 123.78, 119.63, 115.22, 110.67, 100.64, 55.85; **HRMS** (ESI): m/z calcd for $(C_{19}H_{13}NO_3+H)^+$: 304.0974; found: 304.0796.

2-phenyl-4*H*-pyrano[3,2-*c*]pyridin-4-one (5j)

Using 3-iodo-4-hydroxylpyridine and phenylacetylene as substrates, followed the general procedure B and purified by flash chromatography (silica gel, 1:1 petroleum ether/ethyl acetate) to afford 32 mg of **5j** (72%) as a solid.. **^{1}H NMR** (CD_3OD) δ 9.26 (s, 1H), 8.80 (d, *J* = 5.5 Hz, 1H), 8.04 (m, 2H),7.72 (d, *J* = 5.5 Hz, 1H), 7.63-7.55 (m, 3H), 7.01 (s, 1H); **^{13}C NMR** (CD_3OD) δ 177.61, 164.79, 161.49, 152.85, 148.56, 132.14, 130.63, 128.96, 126.34, 119.24, 113.21, 108.48; **HRMS** (ESI): m/z calcd for$(C_{14}H_9NO_2+H)^+$: 224.0706; found: 224.0737.

7-phenyl-5*H*-pyrano[2,3-*d*]pyrimidin-5-one (5k)

Using 5-iodopyrimidin-4-ol and phenylacetylene as substrates, followed the general procedure B and purified by flash chromatography (silica gel, 2:1 petroleum ether/ethyl acetate) to afford 34 mg of **5k** (75%) as a solid. **^{1}H NMR** ($CDCl_3$) δ 9.58 (s, 1H), 9.30 (s, 1H), 8.05 (d, *J* = 7.0 Hz, 2H), 7.65-7.56 (m, 3H), 6.97 (s, 1H); **^{13}C NMR** (DMSO-d_6) δ176.99, 165.84, 164.58, 161.52, 158.83, 132.73, 130.22, 129.36, 126.70, 116.08, 109.63; **HRMS** (ESI): m/z calcd for$(C_{13}H_8N_2O_2+H)^+$: 225.0659; found: 225.0659.

^{1}H-NMR spectra and ^{13}C-NMR spectra

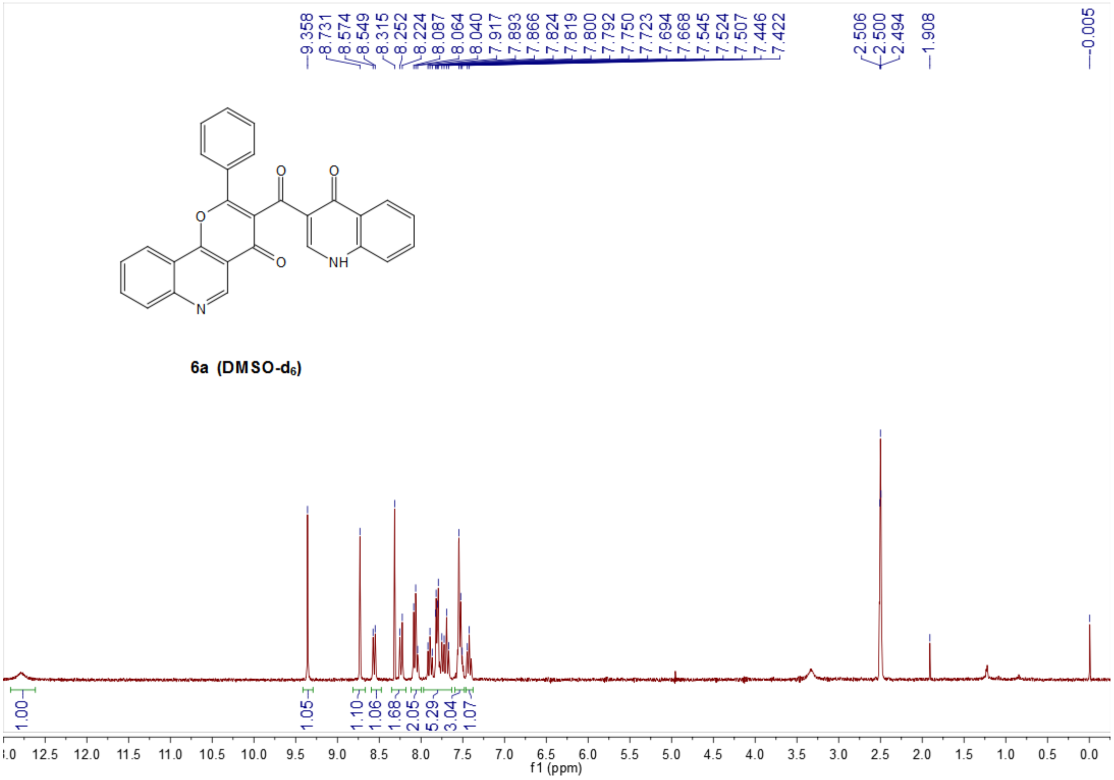

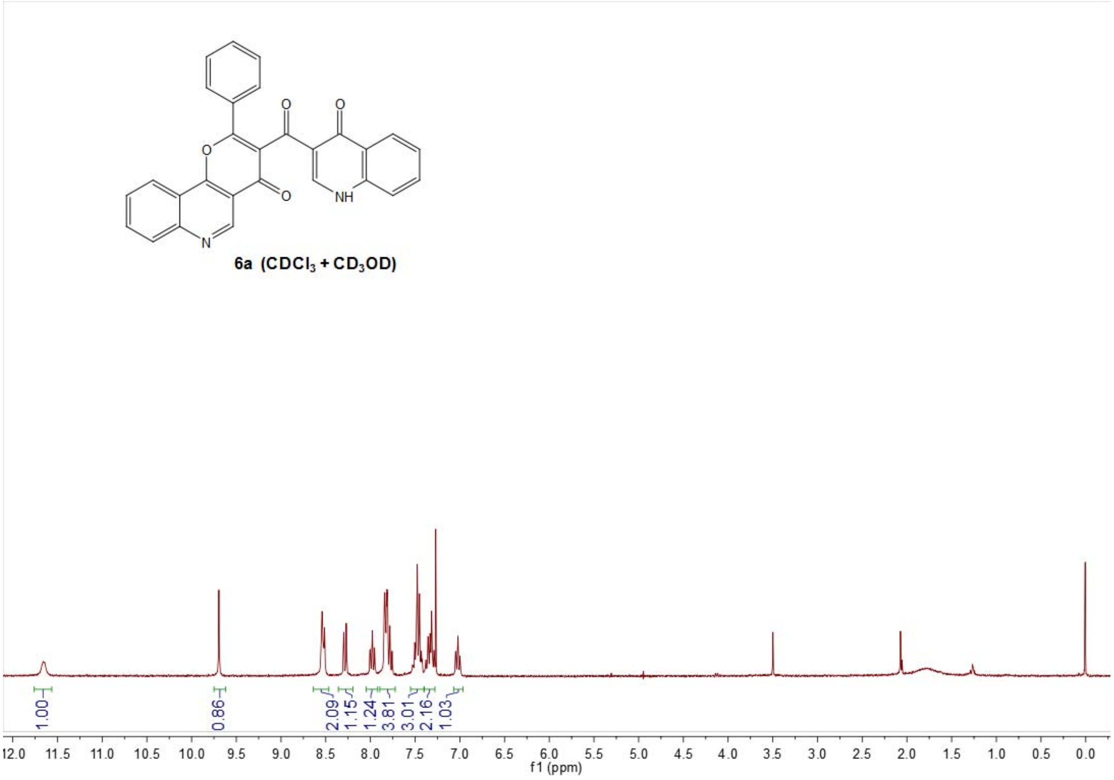

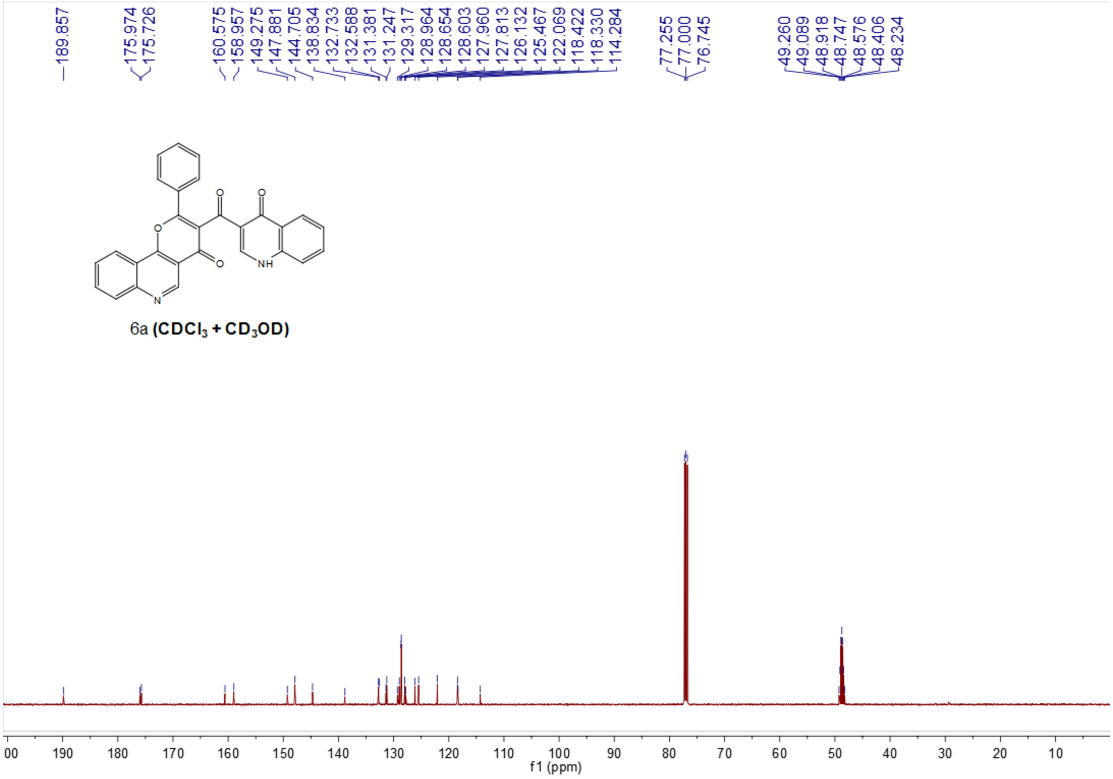
189.857
175.974
175.726
160.575
158.957
149.275
147.881
144.705
138.834
132.733
132.588
131.381
131.247
129.317
128.964
128.654
128.603
127.960
127.813
126.132
125.467
122.069
118.422
118.330
114.284
77.255
77.000
76.745
49.260
49.089
48.918
48.747
48.576
48.406
48.234
6a (CDCl3 + CD3OD)
f1 (ppm)

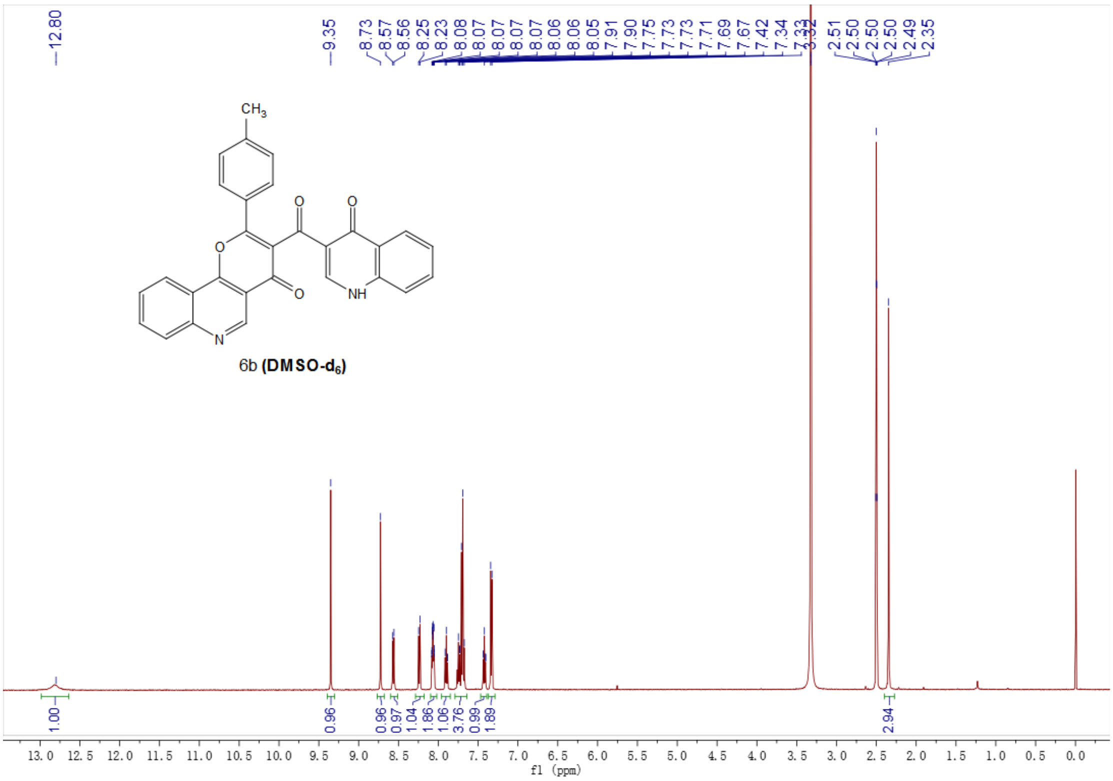
12.80
9.35
8.73
8.57
8.56
8.25
8.23
8.08
8.07
8.07
8.07
8.06
8.05
7.91
7.90
7.75
7.73
7.73
7.71
7.69
7.67
7.42
7.34
7.33
3.32
2.51
2.50
2.50
2.50
2.49
2.35
CH3
O
O
O
O
NH
N
6b (DMSO-d6)
1.00
0.96
0.96
0.97
1.04
1.86
1.06
3.76
0.99
1.89
2.94
13.0 12.5 12.0 11.5 11.0 10.5 10.0 9.5 9.0 8.5 8.0 7.5 7.0 6.5 6.0 5.5 5.0 4.5 4.0 3.5 3.0 2.5 2.0 1.5 1.0 0.5 0.0
f1 (ppm)

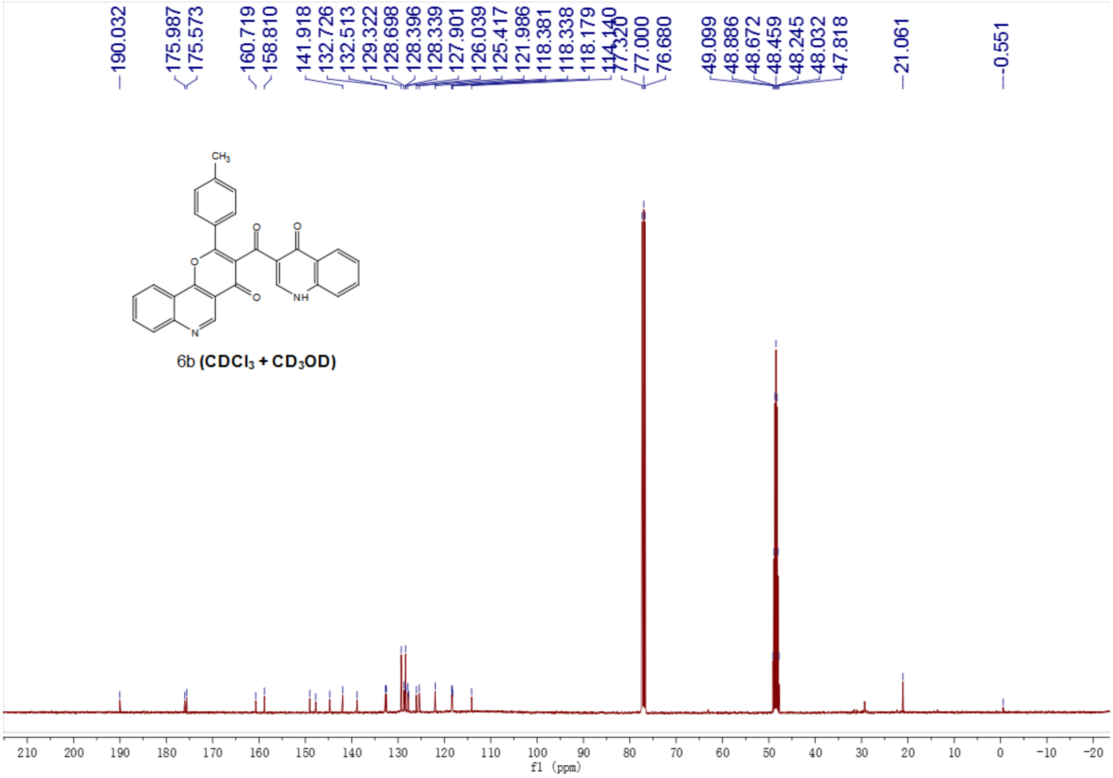
190.032
175.987
175.573
160.719
158.810
141.918
132.726
132.513
129.322
128.698
128.396
128.339
127.901
126.039
125.417
121.986
118.381
118.338
118.179
114.140
77.320
77.000
76.680
49.099
48.886
48.672
48.459
48.245
48.032
47.818
21.061
-0.551
CH3
O
O
O
O
NH
N
6b (CDCl3 + CD3OD)
210 200 190 180 170 160 150 140 130 120 110 100 90 80 70 60 50 40 30 20 10 0 -10 -20
f1 (ppm)

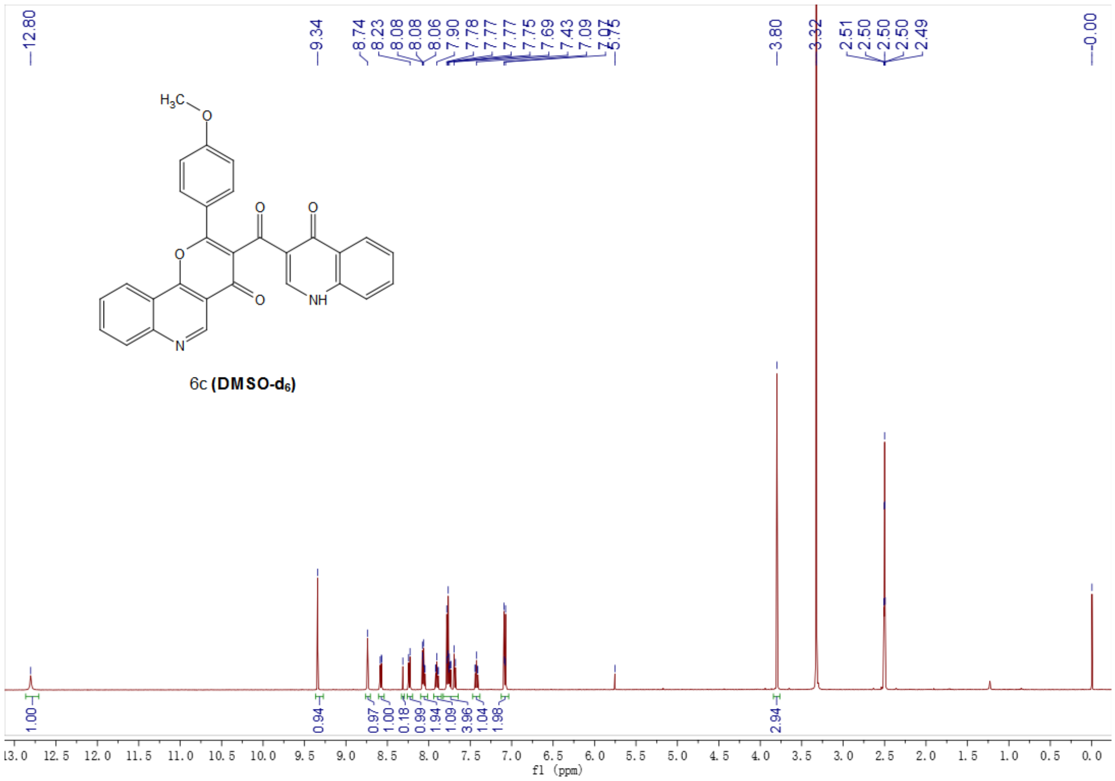
6c (DMSO-d6)
12.80
9.34
8.74 8.23 8.08 8.08 8.06 7.90 7.78 7.77 7.77 7.75 7.69 7.43 7.09 7.07 5.75
3.80
3.32
2.51 2.50 2.50 2.49
0.00
1.00 0.94 0.97 1.00 0.18 0.99 1.94 1.09 3.96 1.04 1.98 2.94
f1 (ppm)

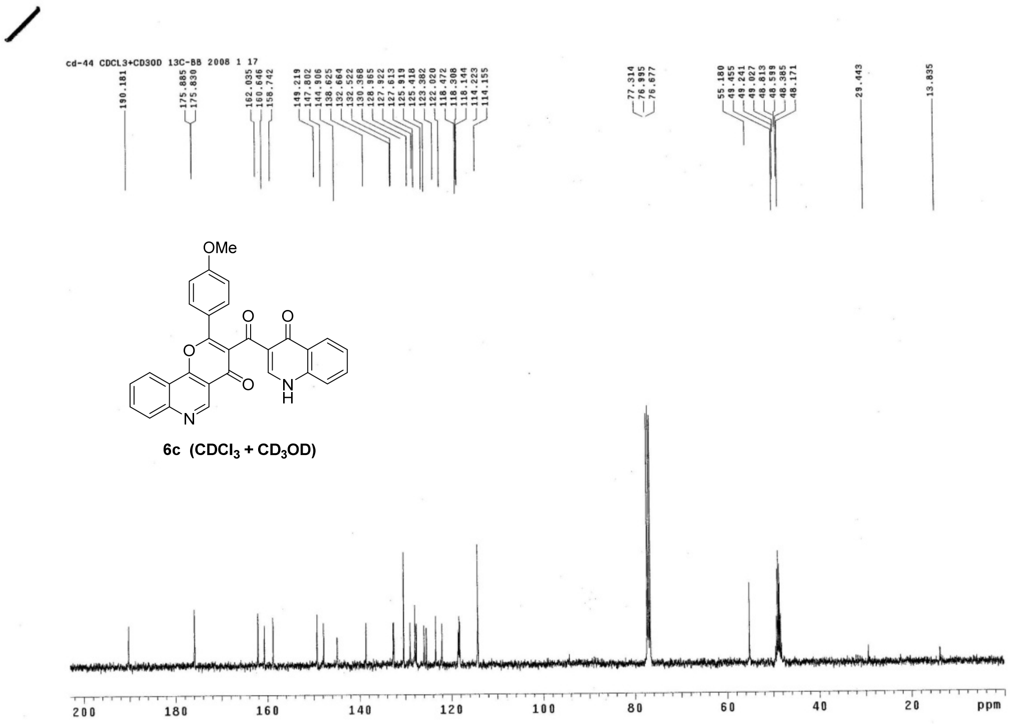
cd-44 CDCL3+CD3OD 13C-BB 2008 1 17
190.181
175.885 175.830
162.035 160.646 158.742
149.219 147.802 144.906 138.625 132.664 130.522 130.368 128.965 127.922 127.613 125.919 125.418 123.382 122.020 118.472 118.308 118.144 114.223 114.155
77.314 76.895 76.877
55.180 49.455 49.241 49.027 48.813 48.599 48.385 48.171
29.443
13.835
6c (CDCl3 + CD3OD)
ppm

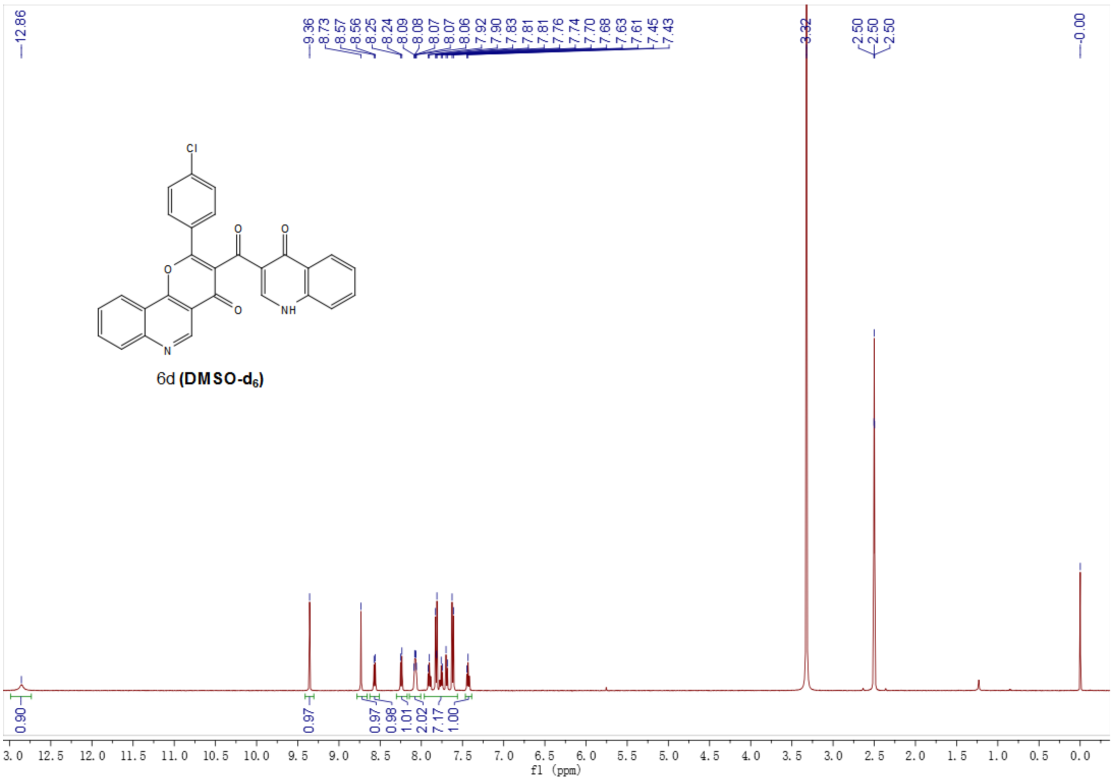
12.86
9.36
8.73
8.57
8.56
8.25
8.24
8.09
8.08
8.07
8.07
8.06
7.92
7.90
7.83
7.81
7.76
7.74
7.70
7.68
7.63
7.61
7.45
7.43
3.32
2.50
2.50
2.50
0.00
Cl
O
O
O
O
NH
N
6d (DMSO-d6)
0.90
0.97
0.97
0.98
1.01
2.02
7.17
1.00
f1 (ppm)

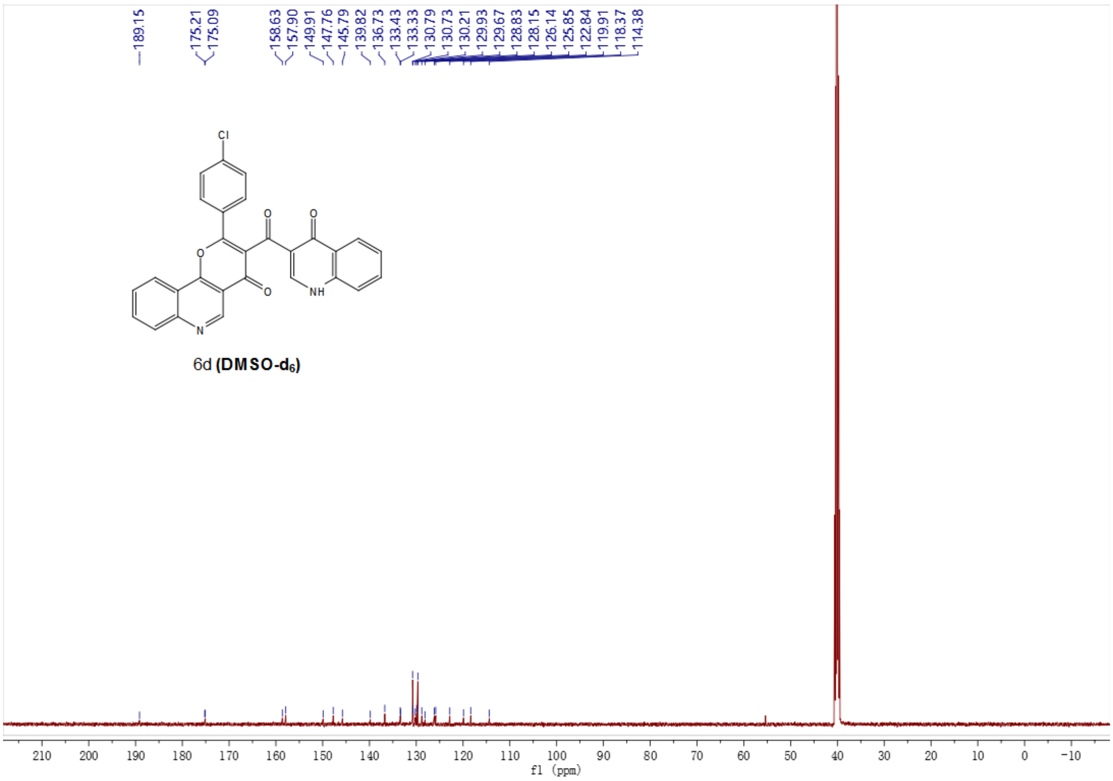
189.15
175.21
175.09
158.63
157.90
149.91
147.76
145.79
139.82
136.73
133.43
133.33
130.79
130.73
130.21
129.93
129.67
128.83
128.15
126.14
125.85
122.84
119.91
118.37
114.38
Cl
O
O
O
O
NH
N
6d (DMSO-d6)
f1 (ppm)

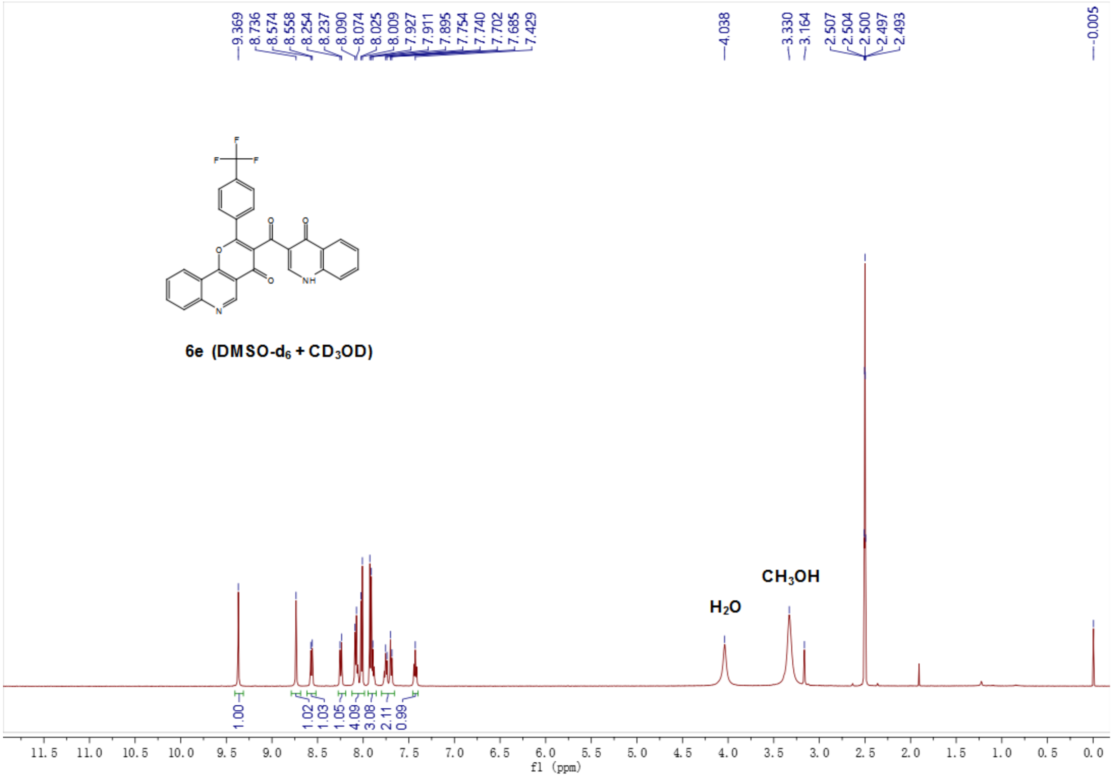
9.369
8.736
8.574
8.558
8.254
8.237
8.090
8.074
8.025
8.009
7.927
7.911
7.895
7.754
7.740
7.702
7.685
7.429
4.038
3.330
3.164
2.507
2.504
2.500
2.497
2.493
-0.005
6e (DMSO-d6 + CD3OD)
H2O
CH3OH
1.00
1.02
1.03
1.05
4.09
3.08
2.11
0.99
f1 (ppm)

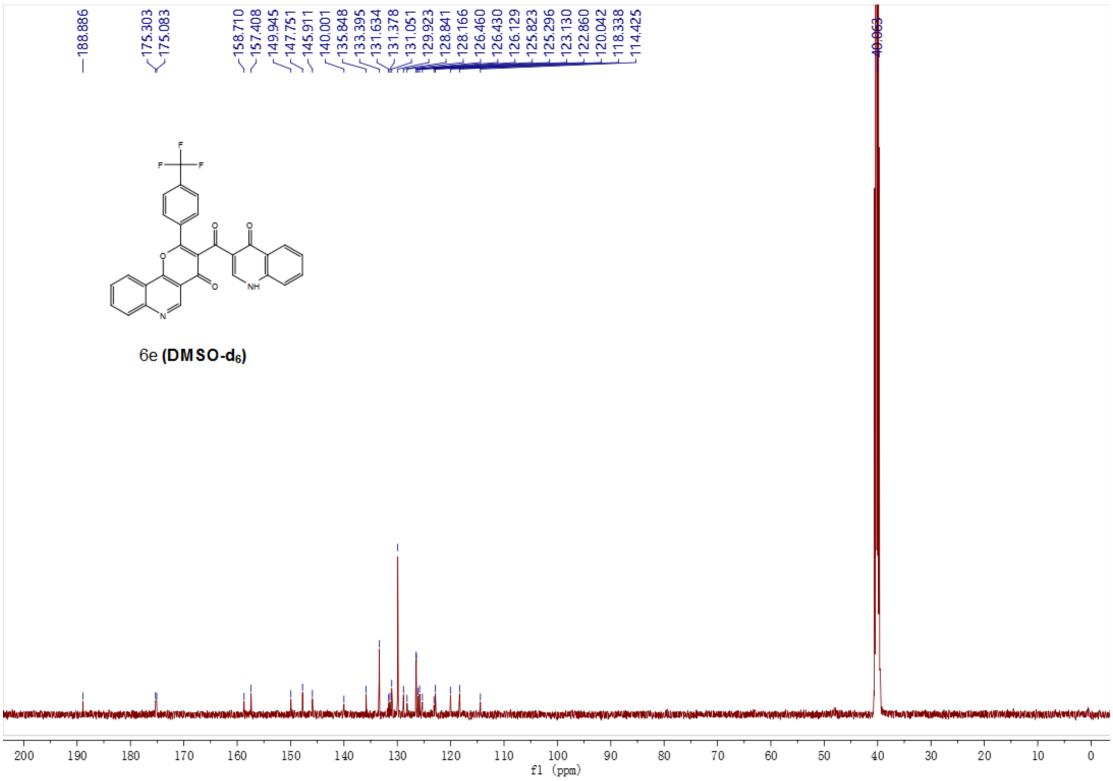
188.886
175.303
175.083
158.710
157.408
149.945
147.751
145.911
140.001
135.848
133.395
131.634
131.378
131.051
129.923
128.841
128.166
126.460
126.430
126.129
125.823
125.296
123.130
122.860
120.042
118.338
114.425
40.063
6e (DMSO-d6)
f1 (ppm)

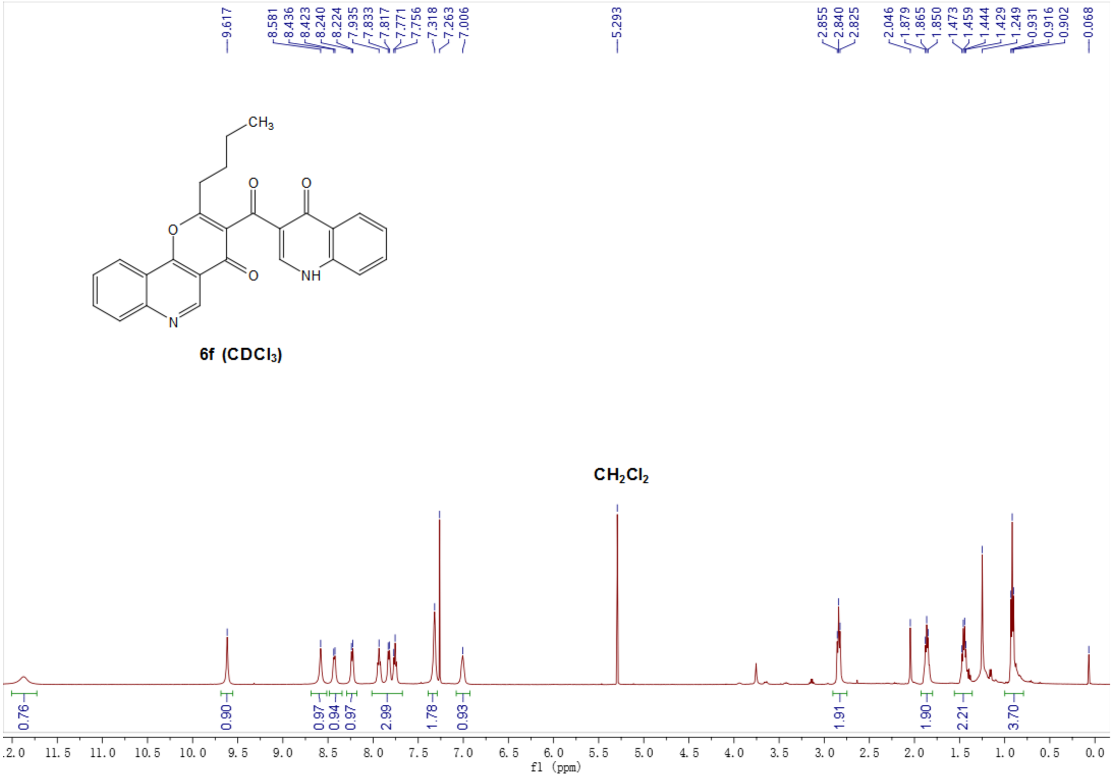
9.617
8.581
8.436
8.423
8.240
8.224
7.935
7.833
7.817
7.771
7.756
7.318
7.263
7.006
5.293
2.855
2.840
2.825
2.046
1.879
1.865
1.850
1.473
1.459
1.444
1.429
1.249
0.931
0.916
0.902
-0.068
CH3
O
O
O
O
NH
N
6f (CDCl3)
CH2Cl2
0.76
0.90
0.97
0.94
0.97
2.99
1.78
0.93
1.91
1.90
2.21
3.70
f1 (ppm)

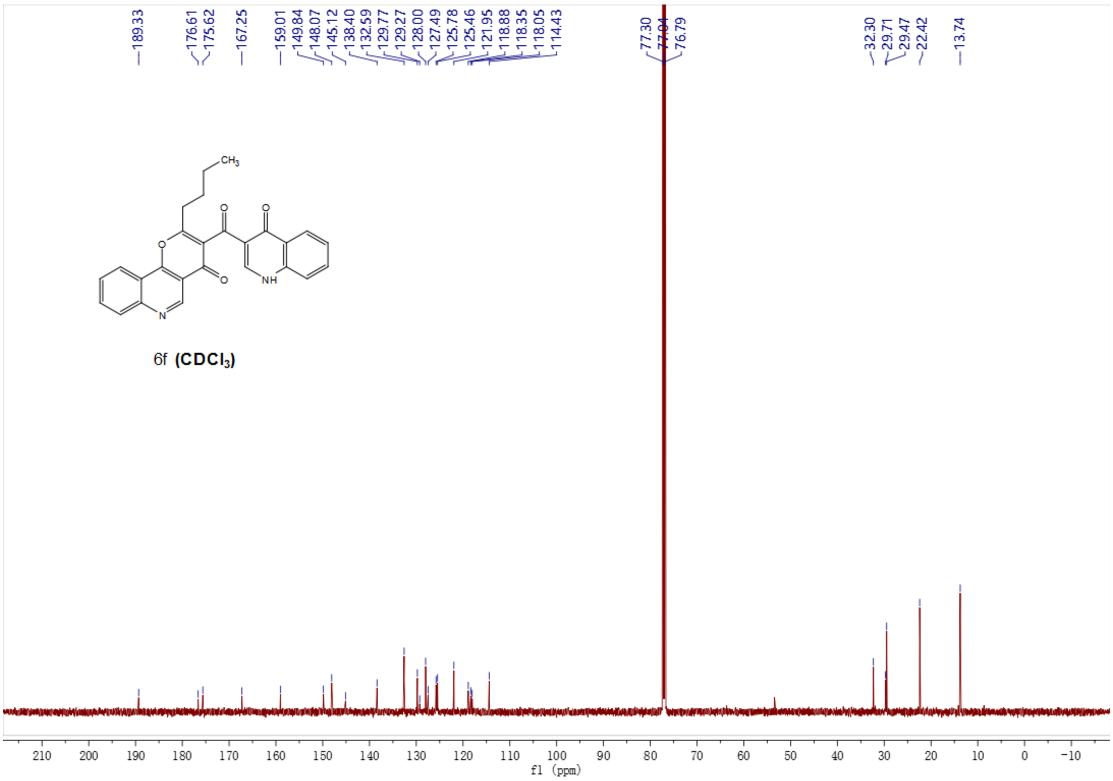
189.33
176.61
175.62
167.25
159.01
149.84
148.07
145.12
138.40
132.59
129.77
129.27
128.00
127.49
125.78
125.46
121.95
118.88
118.35
118.05
114.43
77.30
77.04
76.79
32.30
29.71
29.47
22.42
13.74
CH3
O
O
O
O
NH
N
6f (CDCl3)
f1 (ppm)

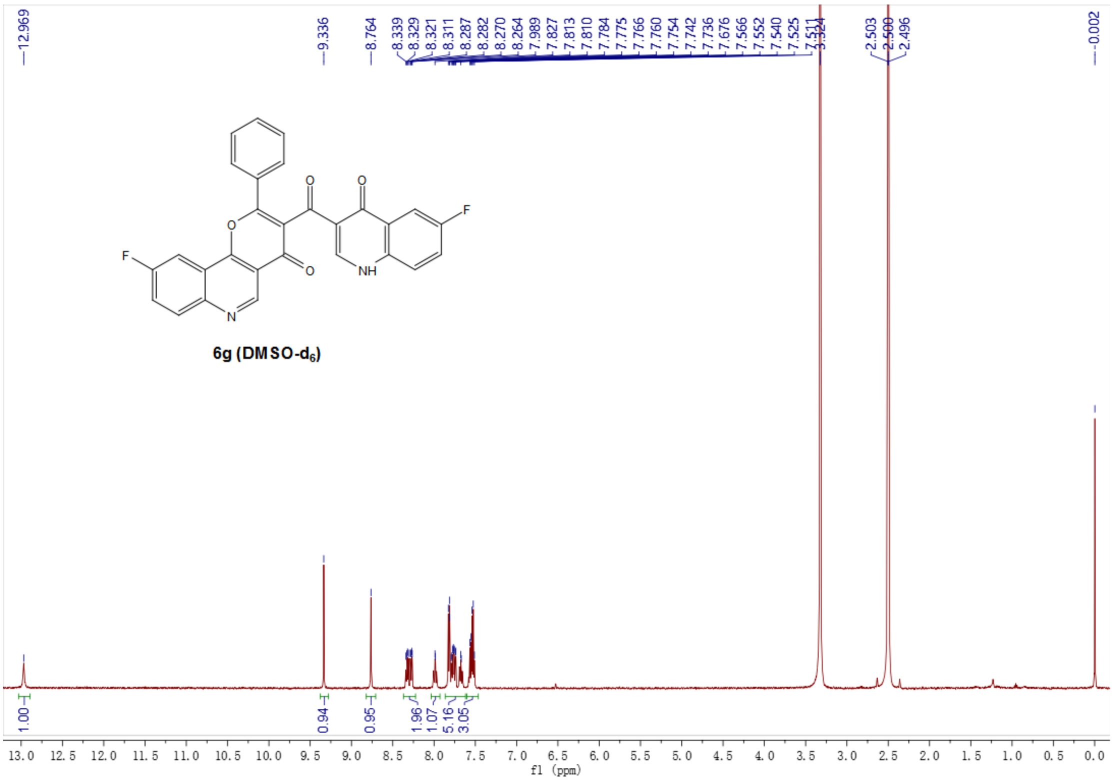
6g (DMSO-d$_6$)
12.969
9.336
8.764
8.339
8.329
8.321
8.311
8.287
8.282
8.270
8.264
7.989
7.827
7.813
7.810
7.784
7.775
7.766
7.760
7.754
7.742
7.736
7.676
7.566
7.552
7.540
7.525
7.511
3.324
2.503
2.496
-0.002
1.00
0.94
0.95
1.96
1.07
5.16
3.05
f1 (ppm)

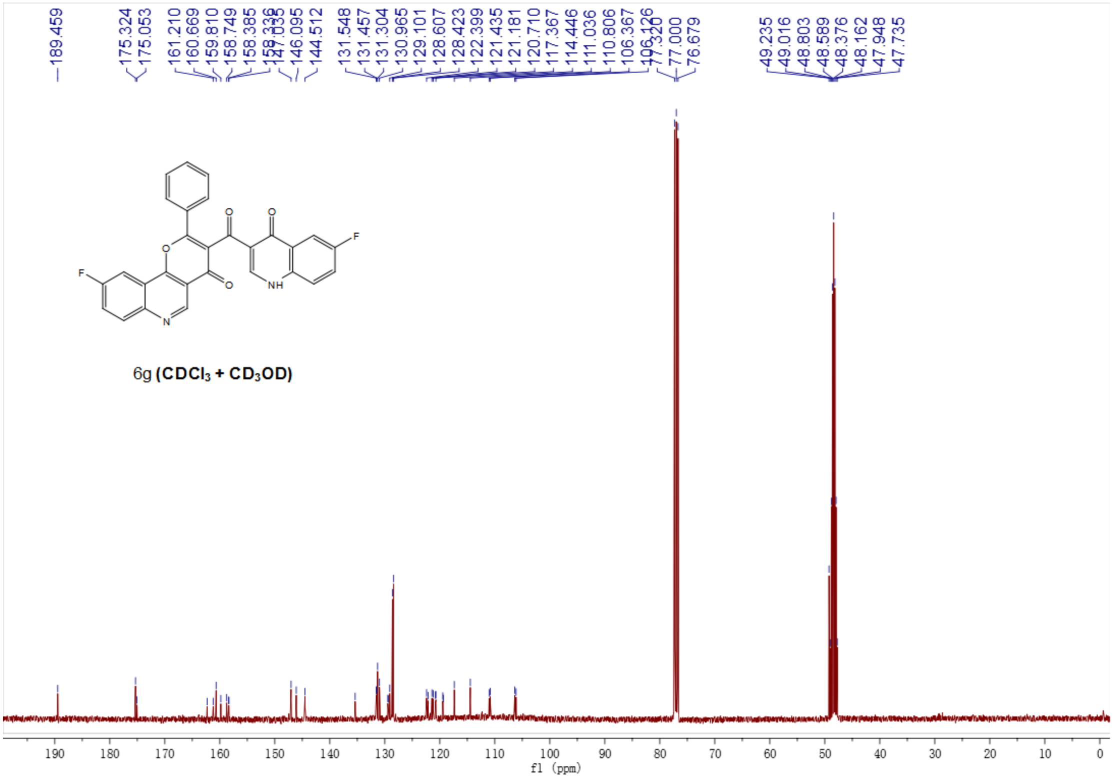
6g (CDCl$_3$ + CD$_3$OD)
189.459
175.324
175.053
161.210
160.669
159.810
158.749
158.385
146.095
144.512
131.548
131.457
131.304
130.965
129.101
128.607
128.423
122.399
121.435
121.181
120.710
117.367
114.446
111.036
110.806
106.367
77.000
76.679
49.235
49.016
48.803
48.589
48.376
48.162
47.948
47.735
f1 (ppm)

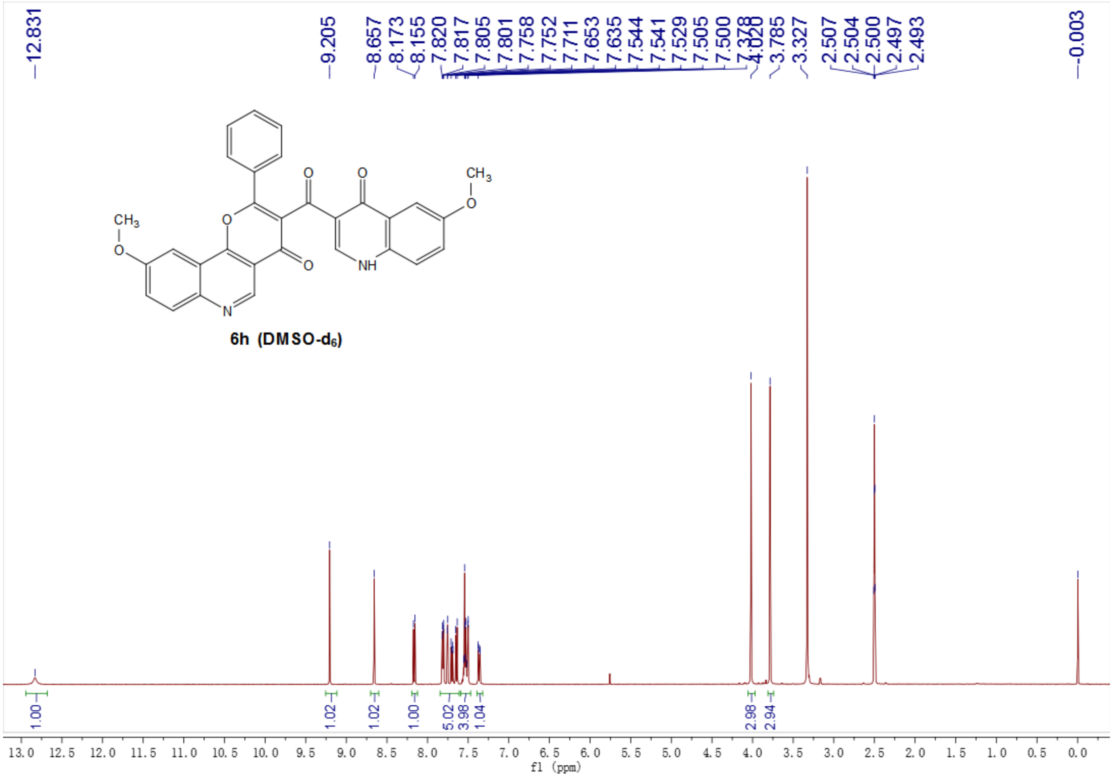
12.831
9.205
8.657
8.173
8.155
7.820
7.817
7.805
7.801
7.758
7.752
7.711
7.653
7.635
7.544
7.541
7.529
7.505
7.500
3.785
3.327
2.507
2.504
2.500
2.497
2.493
-0.003
6h (DMSO-d6)
1.00
1.02
1.02
1.00
5.02
3.98
1.04
2.98
2.94
f1 (ppm)

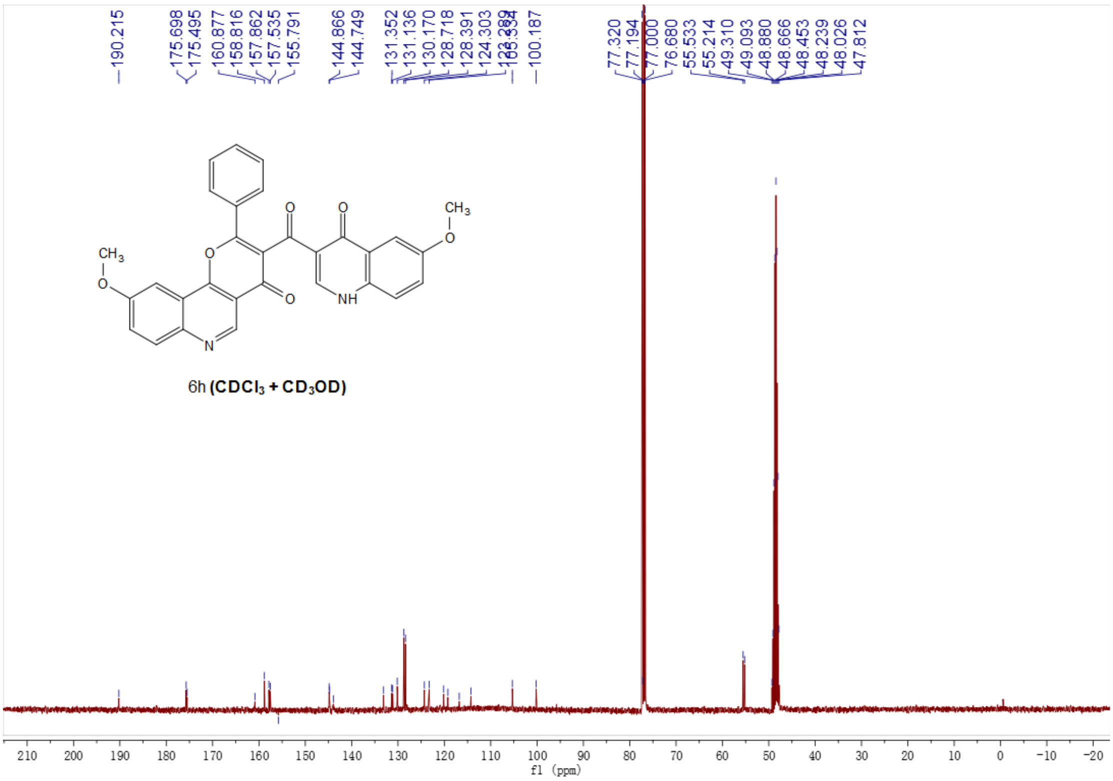
190.215
175.698
175.495
160.877
158.816
157.862
157.535
155.791
144.866
144.749
131.352
131.136
130.170
128.718
128.391
124.303
100.187
77.320
77.194
77.000
76.680
55.533
55.214
49.310
49.093
48.880
48.666
48.453
48.239
48.026
47.812
6h (CDCl3 + CD3OD)
f1 (ppm)

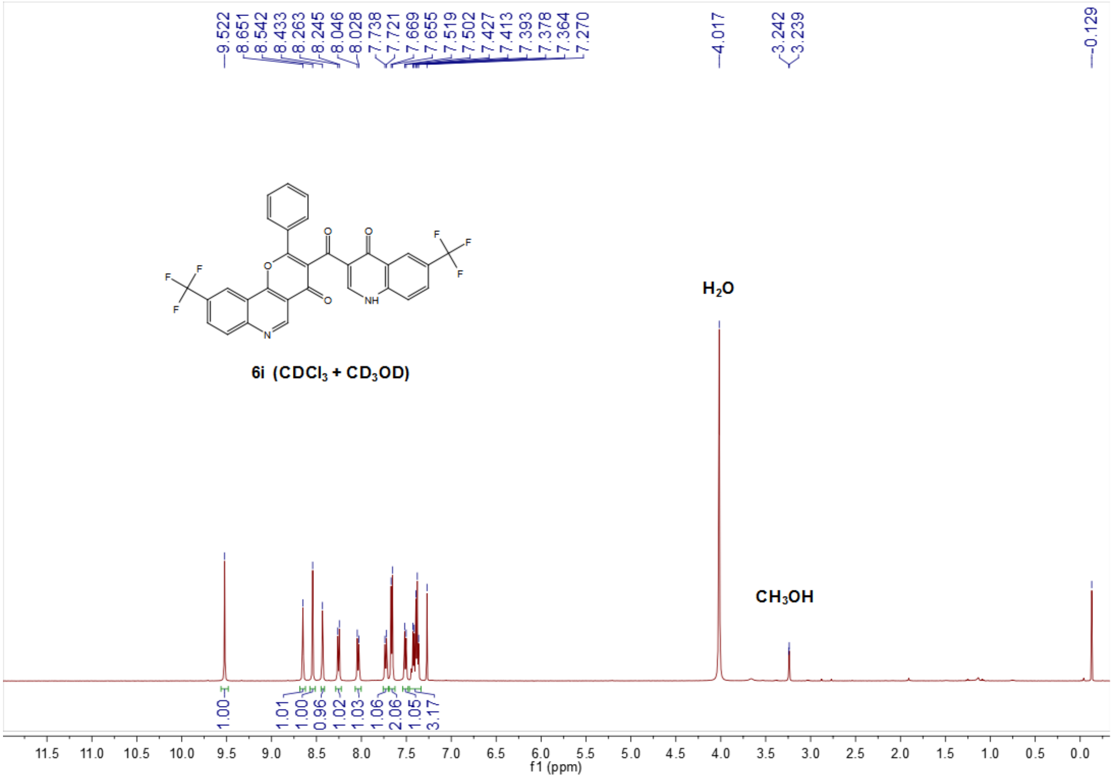
9.522
8.651
8.542
8.433
8.263
8.245
8.046
8.028
7.738
7.721
7.669
7.655
7.519
7.502
7.427
7.413
7.393
7.378
7.364
7.270
4.017
3.242
3.239
-0.129
6i (CDCl3 + CD3OD)
H2O
CH3OH
1.00
1.01
1.00
0.96
1.02
1.03
1.06
2.06
1.05
3.17
f1 (ppm)

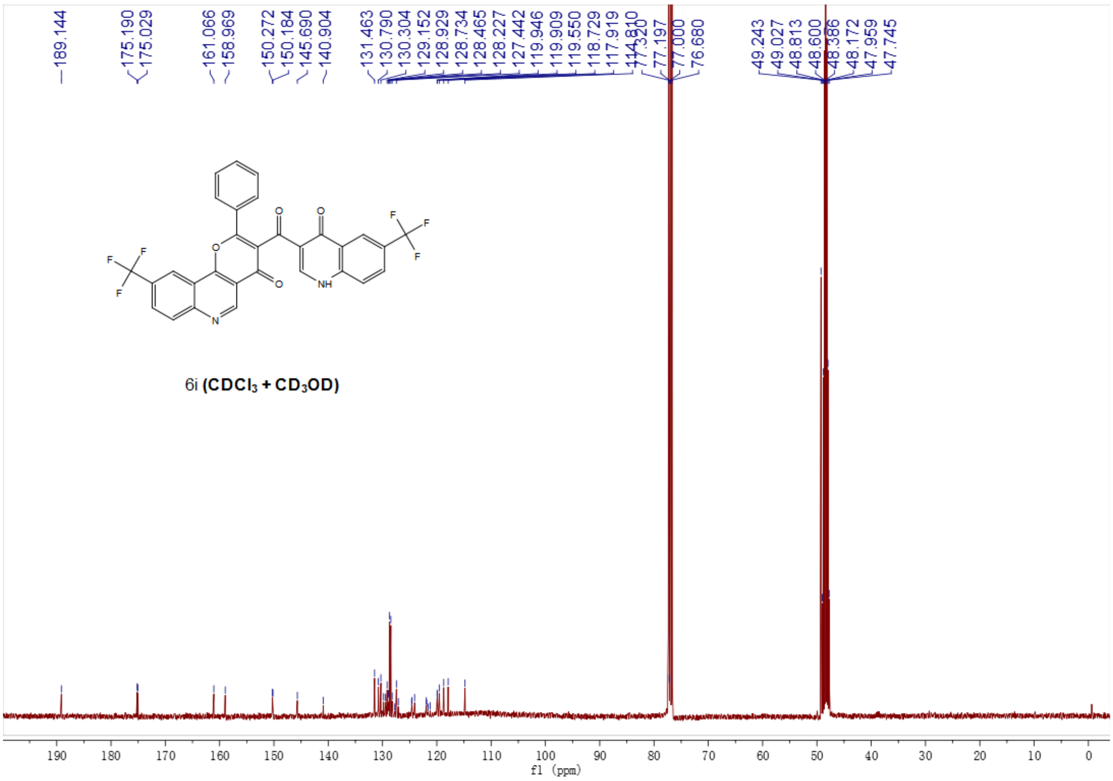
189.144
175.190
175.029
161.066
158.969
150.272
150.184
145.690
140.904
131.463
130.790
130.304
129.152
128.929
128.734
128.465
128.227
127.442
119.946
119.909
119.550
118.729
117.919
114.810
77.320
77.197
77.000
76.680
49.243
49.027
48.813
48.600
48.386
48.172
47.959
47.745
6i (CDCl3 + CD3OD)
f1 (ppm)

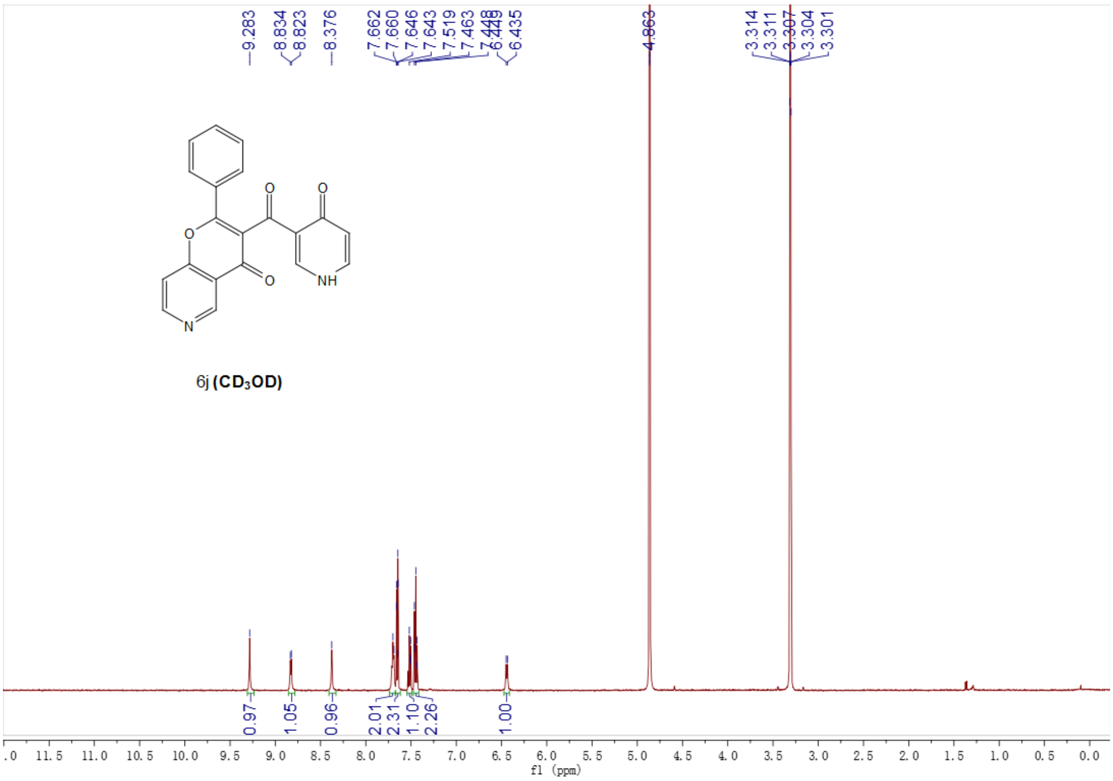

9.283
8.834
8.823
8.376
7.662
7.660
7.646
7.643
7.519
7.463
6.435
4.863
3.314
3.311
3.307
3.304
3.301
6j (CD3OD)
0.97
1.05
0.96
2.01
2.31
1.10
2.26
1.00
f1 (ppm)

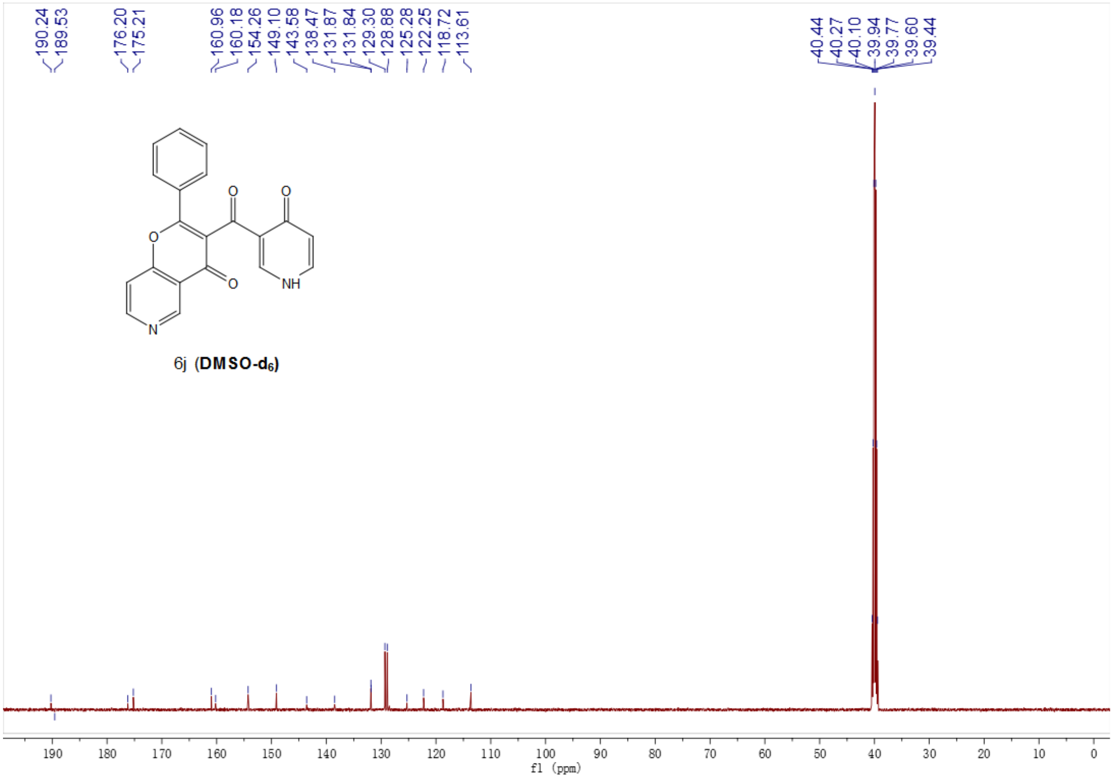

190.24
189.53
176.20
175.21
160.96
160.18
154.26
149.10
143.58
138.47
131.87
131.84
129.30
128.88
125.28
122.25
118.72
113.61
40.44
40.27
40.10
39.94
39.77
39.60
39.44
6j (DMSO-d6)
f1 (ppm)

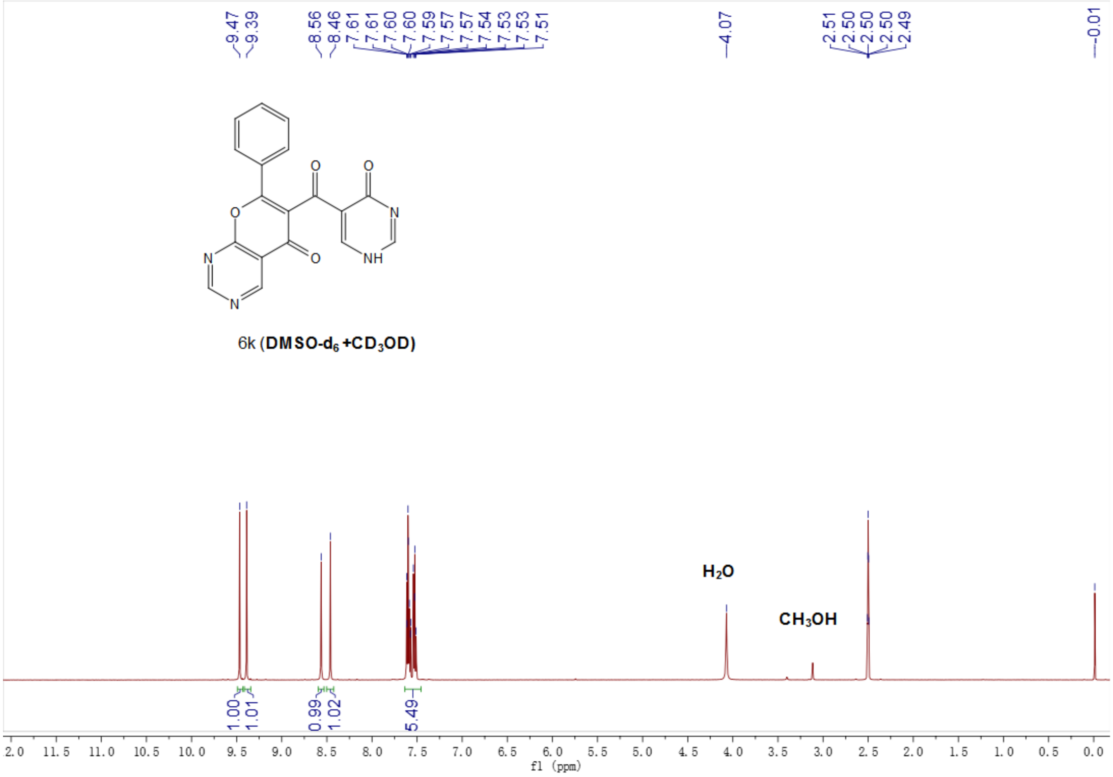
9.47
9.39
8.56
8.46
7.61
7.61
7.60
7.60
7.59
7.57
7.57
7.54
7.53
7.53
7.51
4.07
2.51
2.50
2.50
2.50
2.49
0.01
6k (DMSO-d6 +CD3OD)
H2O
CH3OH
1.00
1.01
0.99
1.02
5.49
f1 (ppm)

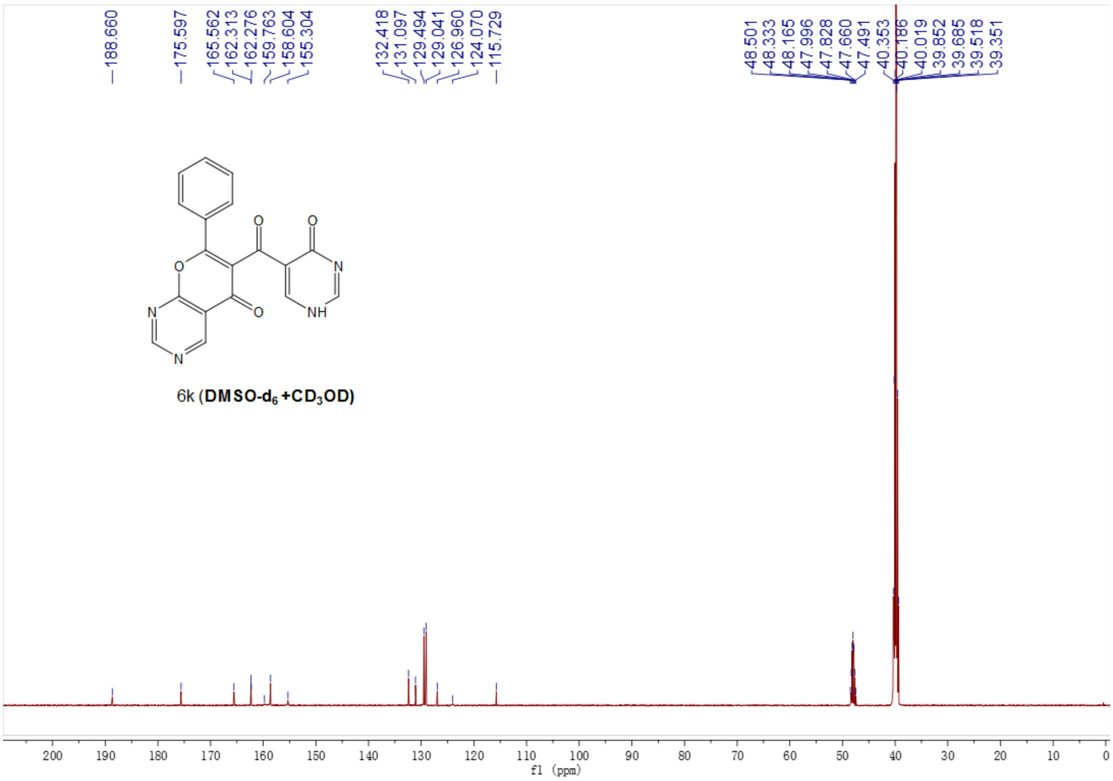
188.660
175.597
165.562
162.313
162.276
159.763
158.604
155.304
132.418
131.097
129.494
129.041
126.960
124.070
115.729
48.501
48.333
48.165
47.996
47.828
47.660
47.491
40.353
40.186
40.019
39.852
39.685
39.518
39.351
6k (DMSO-d6 +CD3OD)
f1 (ppm)

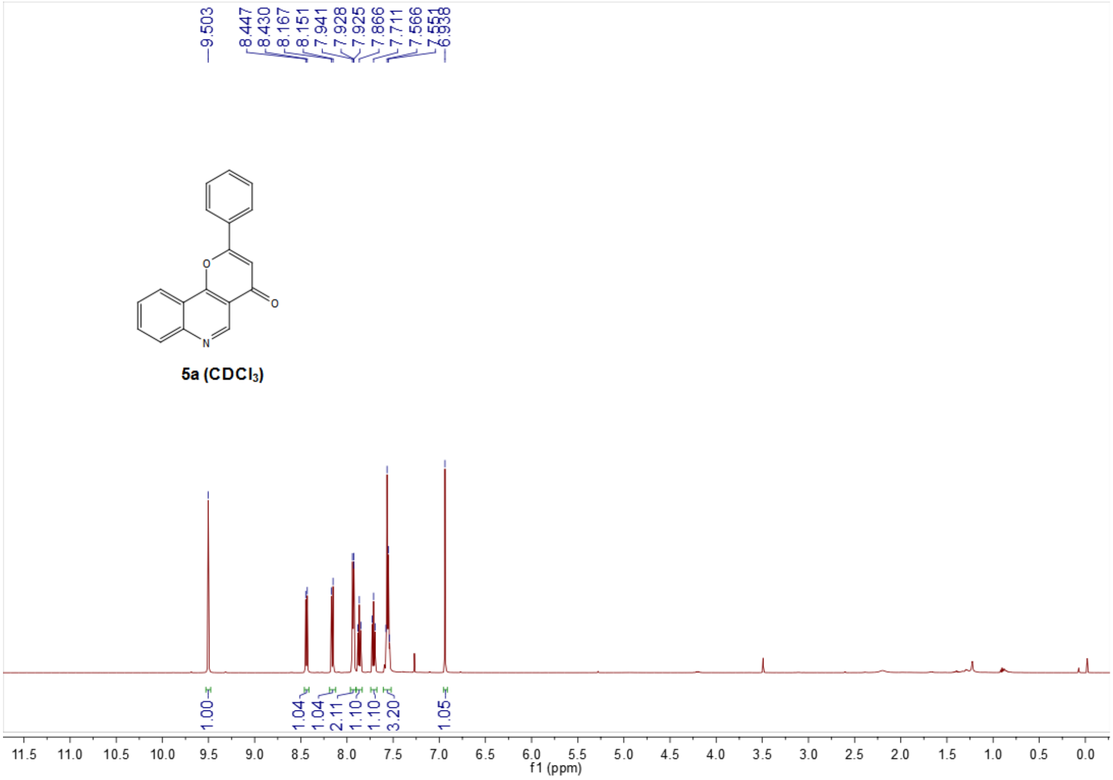
9.503
8.447
8.430
8.167
8.151
7.941
7.928
7.925
7.866
7.711
7.566
7.551
6.938
5a (CDCl3)
1.00
1.04
1.04
2.11
1.10
1.10
3.20
1.05
f1 (ppm)

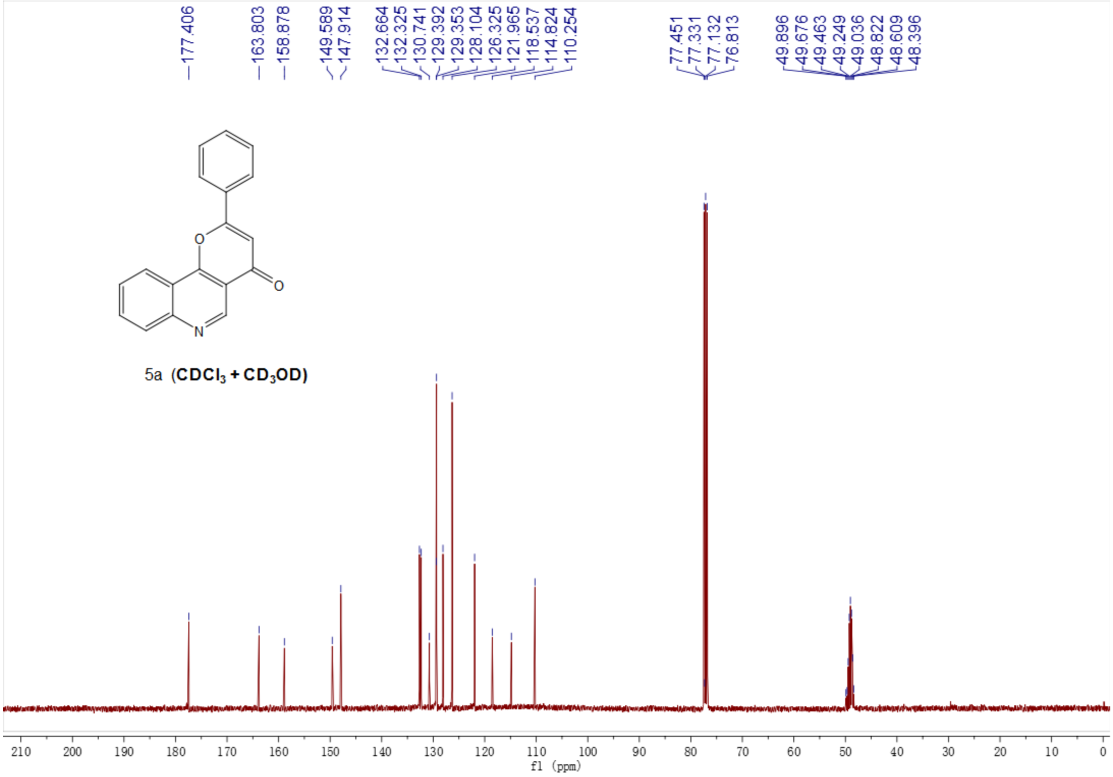
177.406
163.803
158.878
149.589
147.914
132.664
132.325
130.741
129.392
129.353
128.104
126.325
121.965
118.537
114.824
110.254
77.451
77.331
77.132
76.813
49.896
49.676
49.463
49.249
49.036
48.822
48.609
48.396
5a (CDCl3 + CD3OD)
f1 (ppm)

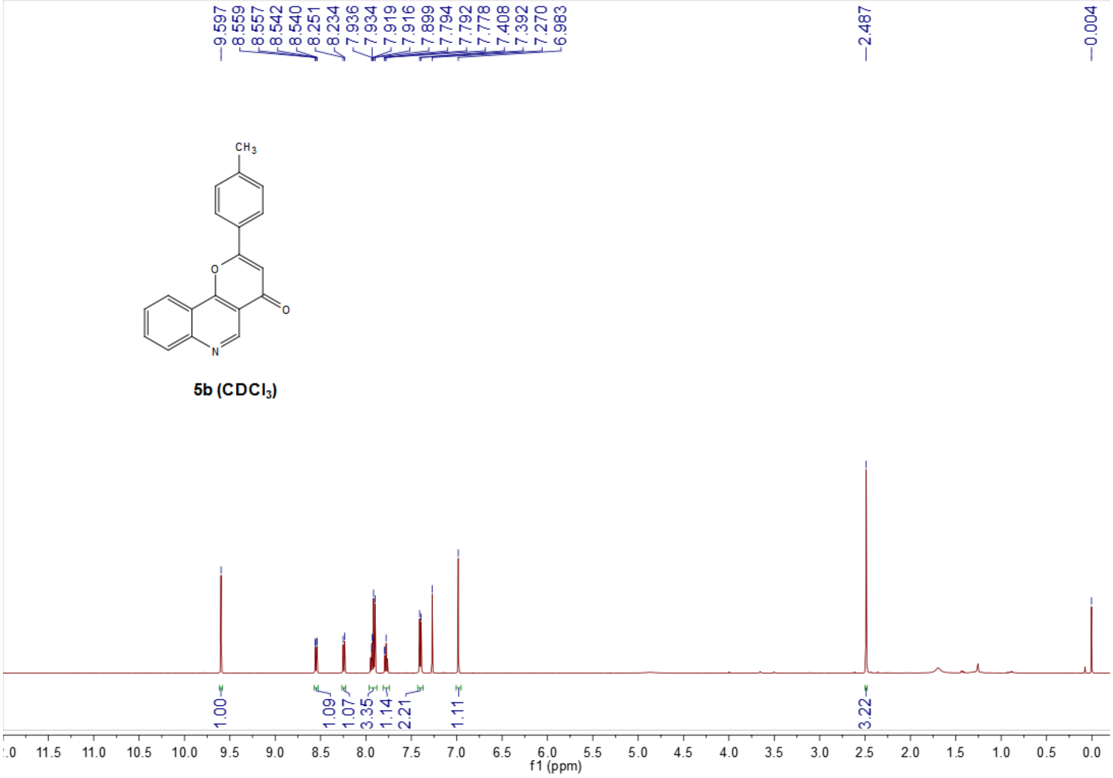
9.597 8.559 8.557 8.542 8.540 8.251 8.234 7.936 7.934 7.919 7.916 7.899 7.794 7.792 7.778 7.408 7.392 7.270 6.983
2.487
-0.004
CH_3
O
O
N
5b ($CDCl_3$)
1.00 1.09 1.07 3.35 1.14 2.21 1.11 3.22
f1 (ppm)

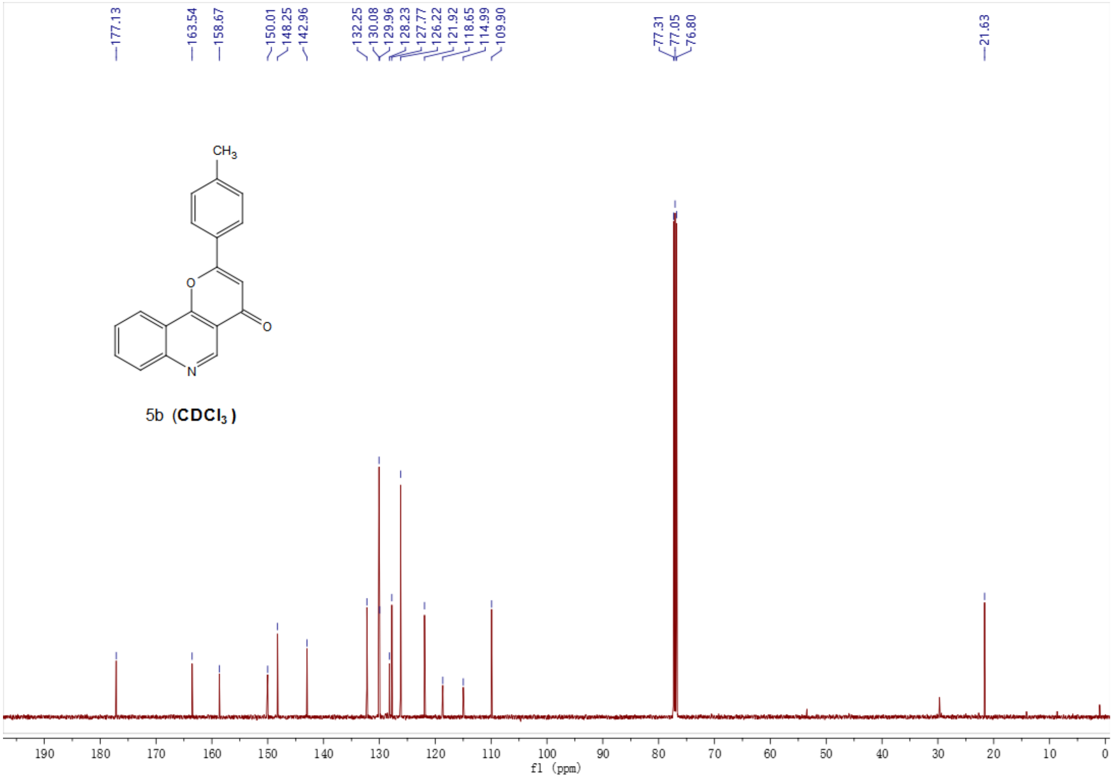
177.13 163.54 158.67 150.01 148.25 142.96 132.25 130.08 129.96 128.23 127.77 126.22 121.92 118.65 114.99 109.90
77.31 77.05 76.80
21.63
CH_3
O
O
N
5b ($CDCl_3$)
f1 (ppm)

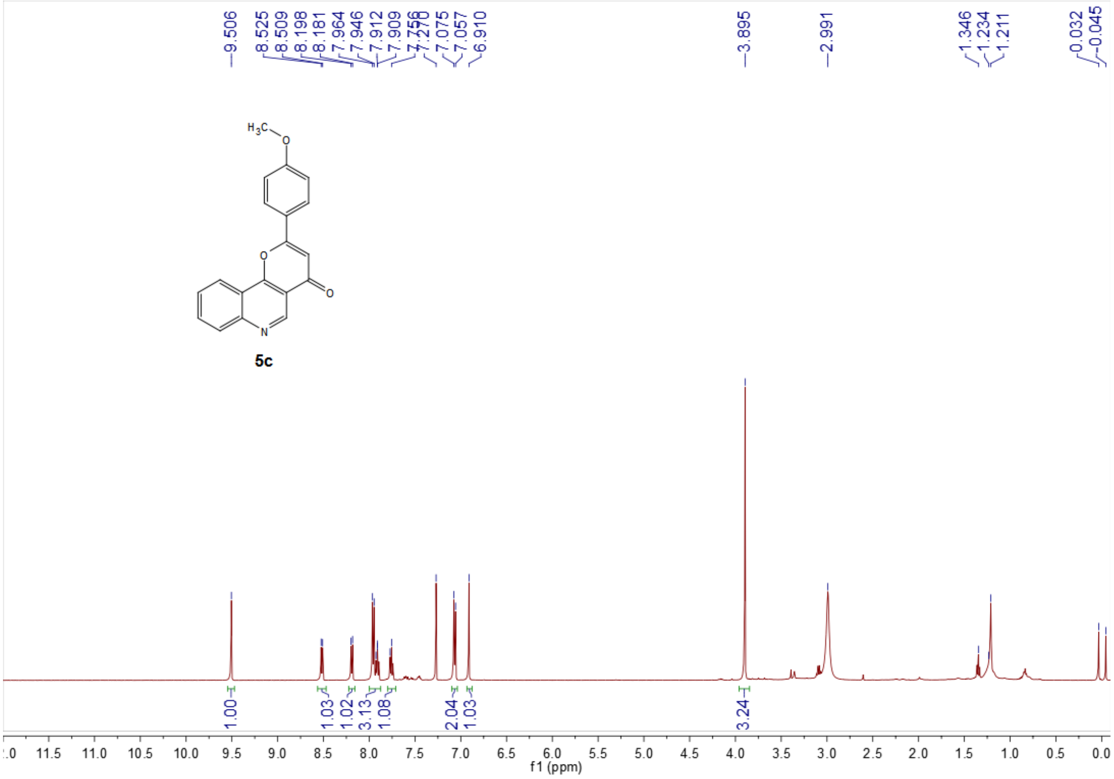
5c
9.506
8.525
8.509
8.198
8.181
7.964
7.946
7.912
7.909
7.270
7.075
7.057
6.910
3.895
2.991
1.346
1.234
1.211
0.032
-0.045
1.00
1.03
1.02
3.13
1.08
2.04
1.03
3.24
f1 (ppm)

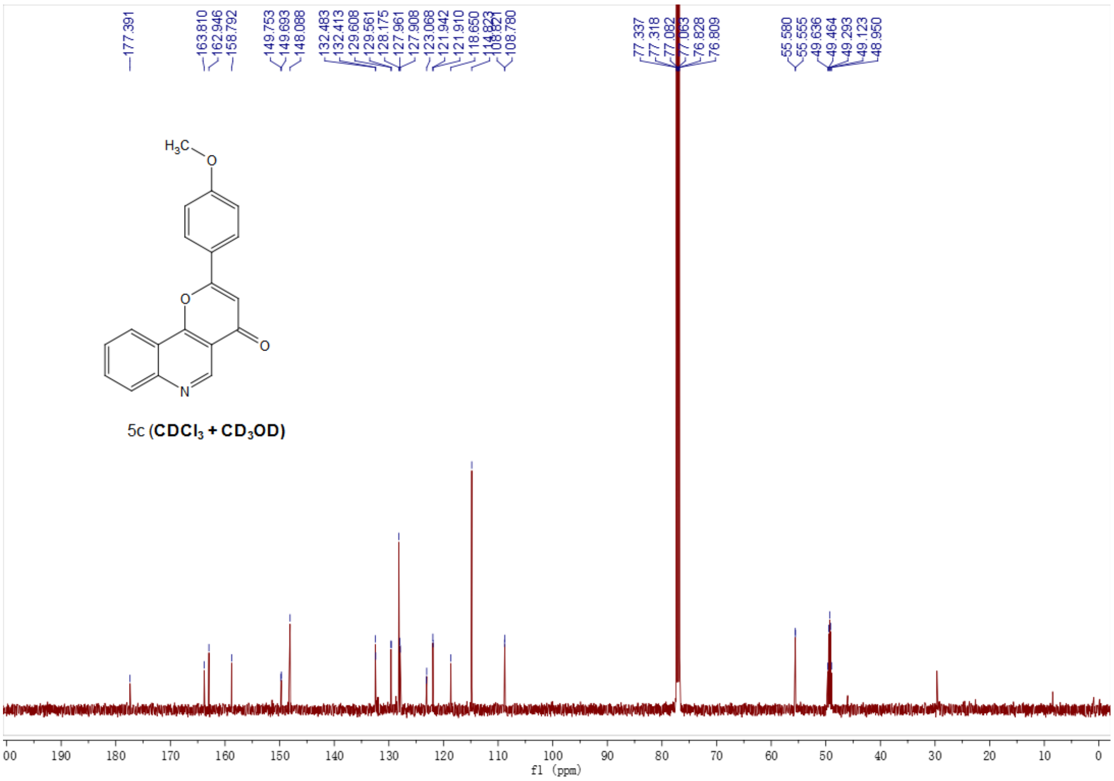
5c (CDCl3 + CD3OD)
177.391
163.810
162.946
158.792
149.753
149.693
148.088
132.483
132.413
129.608
129.561
128.175
127.961
127.908
123.068
121.942
121.910
118.650
108.780
77.337
77.318
77.082
77.063
76.828
76.809
55.580
55.555
49.636
49.464
49.293
49.123
48.950
f1 (ppm)

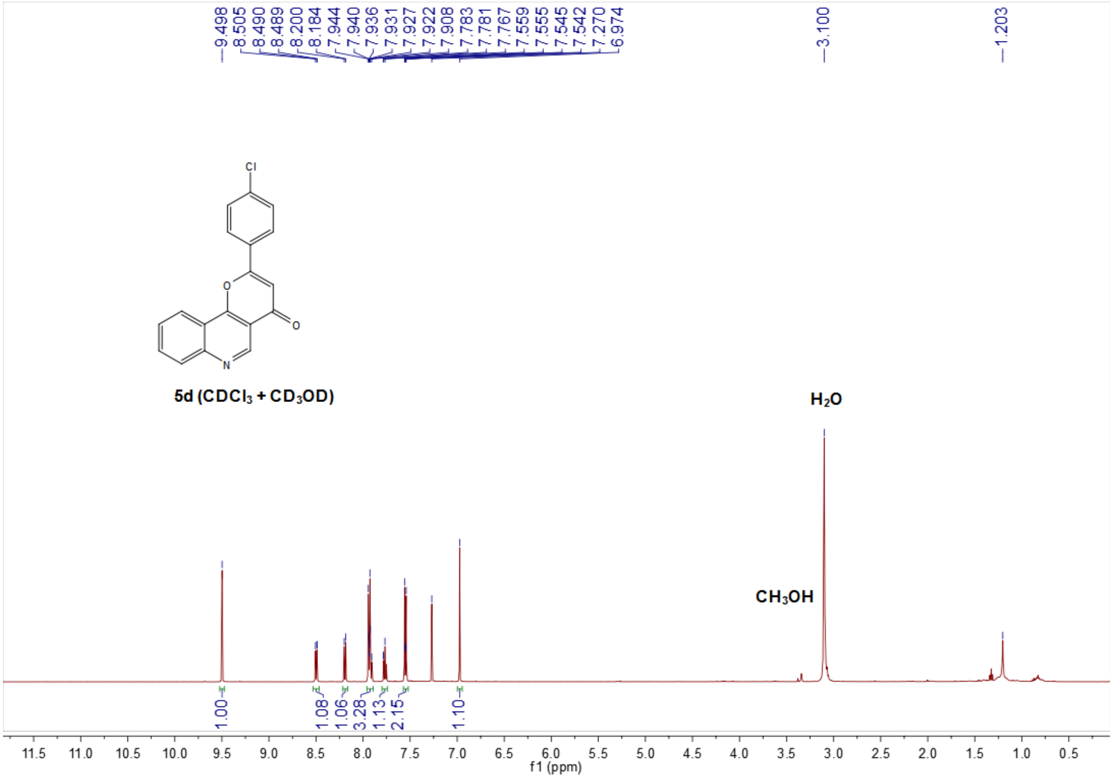

9.498
8.505
8.490
8.489
8.200
8.184
7.944
7.940
7.936
7.931
7.927
7.922
7.908
7.783
7.781
7.767
7.559
7.555
7.545
7.542
7.270
6.974
3.100
1.203
Cl
O
O
N
5d (CDCl3 + CD3OD)
H2O
CH3OH
1.00
1.08
1.06
3.28
1.13
2.15
1.10
f1 (ppm)

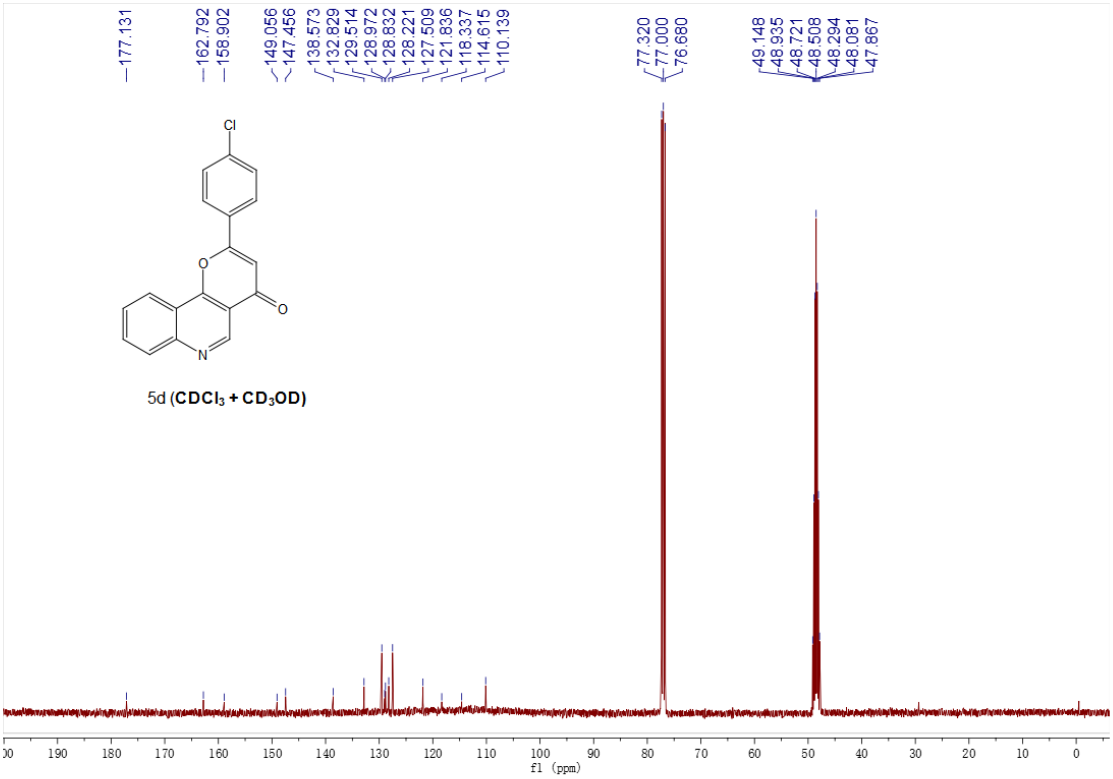

177.131
162.792
158.902
149.056
147.456
138.573
132.829
129.514
128.972
128.832
128.221
127.509
121.836
118.337
114.615
110.139
77.320
77.000
76.680
49.148
48.935
48.721
48.508
48.294
48.081
47.867
Cl
O
O
N
5d (CDCl3 + CD3OD)
f1 (ppm)

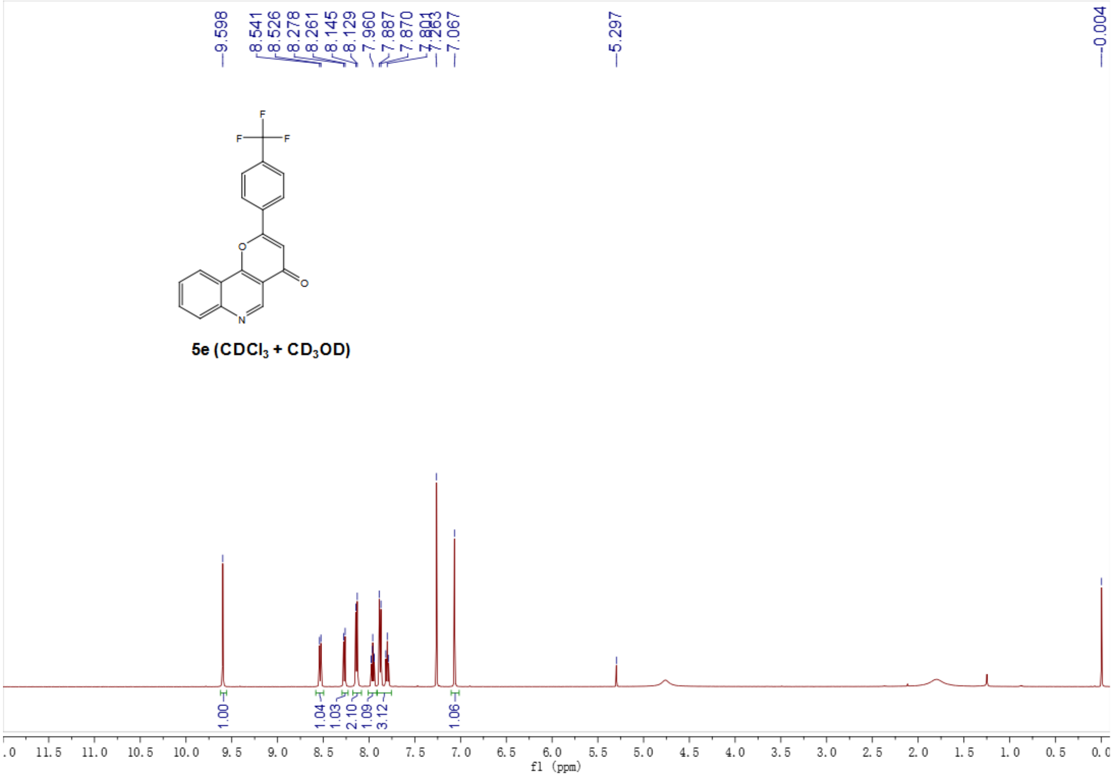
9.598
8.541
8.526
8.278
8.261
8.145
8.129
7.960
7.887
7.870
7.263
7.067
5.297
0.004
5e (CDCl3 + CD3OD)
1.00
1.04
1.03
2.10
1.09
3.12
1.06
fl (ppm)

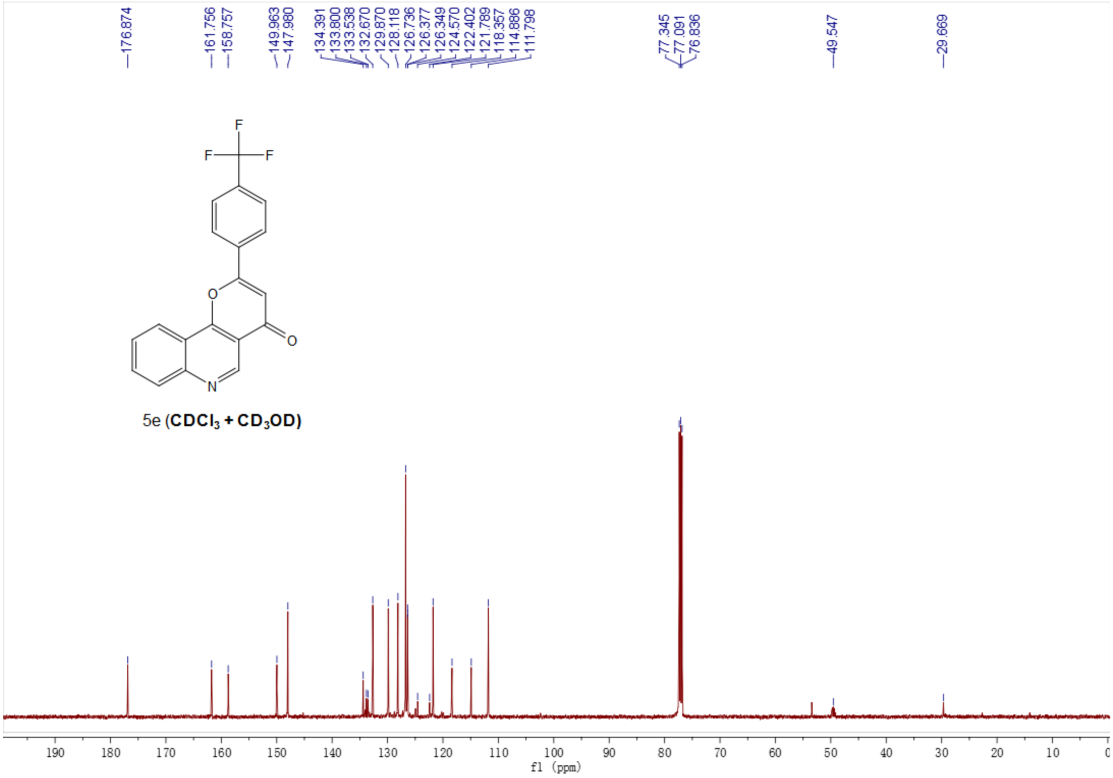
176.874
161.756
158.757
149.963
147.980
134.391
133.800
133.538
132.670
129.870
128.118
126.736
126.377
126.349
124.570
122.402
121.789
118.357
114.886
111.798
77.345
77.091
76.836
49.547
29.669
5e (CDCl3 + CD3OD)
fl (ppm)

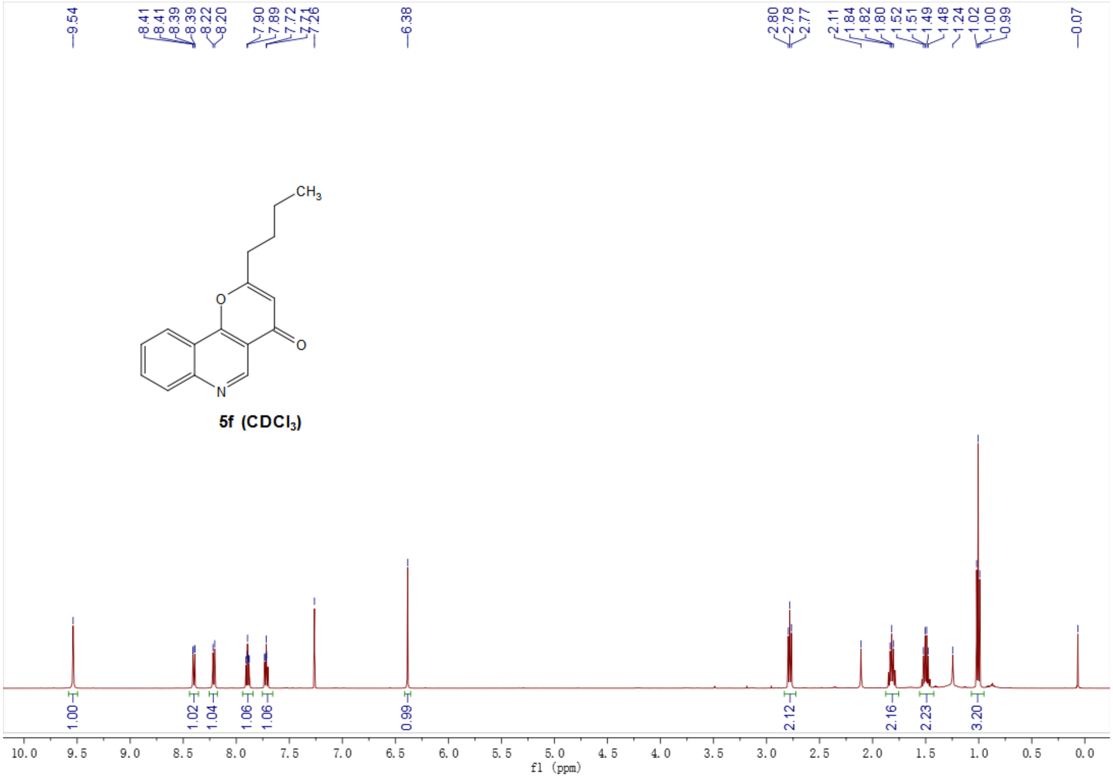
9.54
8.41
8.41
8.39
8.39
8.22
8.20
7.90
7.89
7.72
7.71
7.26
6.38
2.80
2.78
2.77
2.11
1.84
1.82
1.80
1.52
1.51
1.49
1.48
1.24
1.02
1.00
0.99
0.07
CH3
O
O
N
5f (CDCl3)
1.00
1.02
1.04
1.06
1.06
0.99
2.12
2.16
2.23
3.20
f1 (ppm)

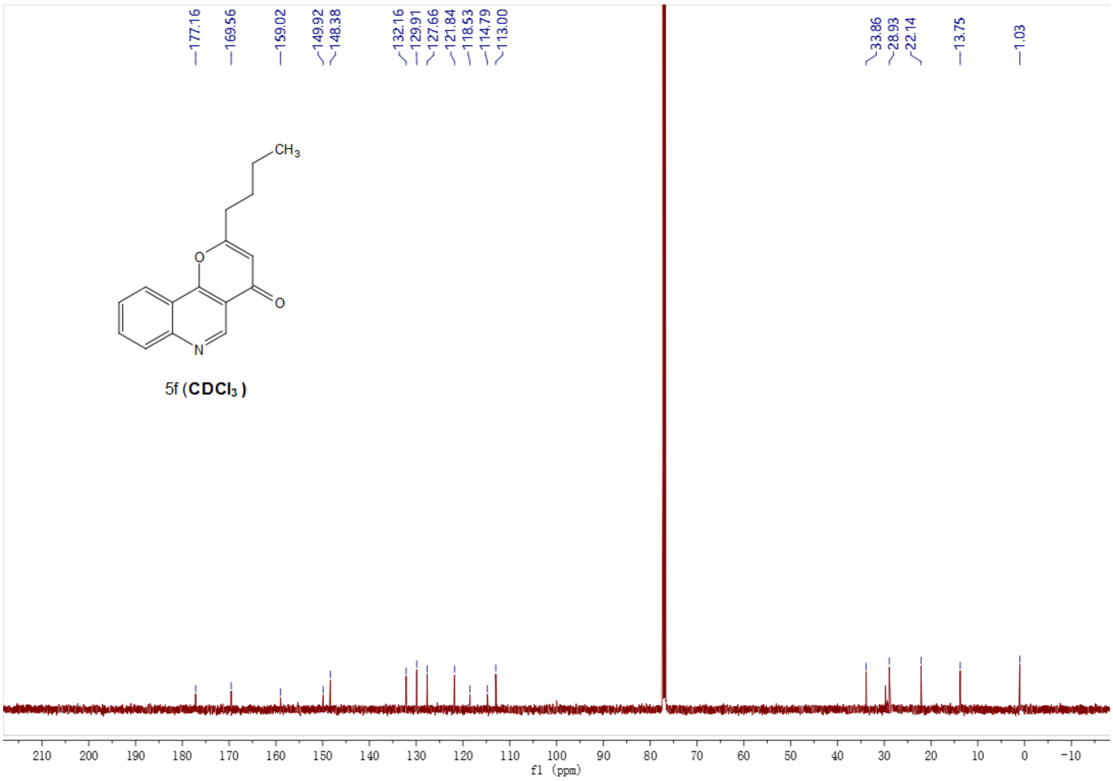
177.16
169.56
159.02
149.92
148.38
132.16
129.91
127.66
121.84
118.53
114.79
113.00
33.86
28.93
22.14
13.75
1.03
CH3
O
O
N
5f (CDCl3)
f1 (ppm)

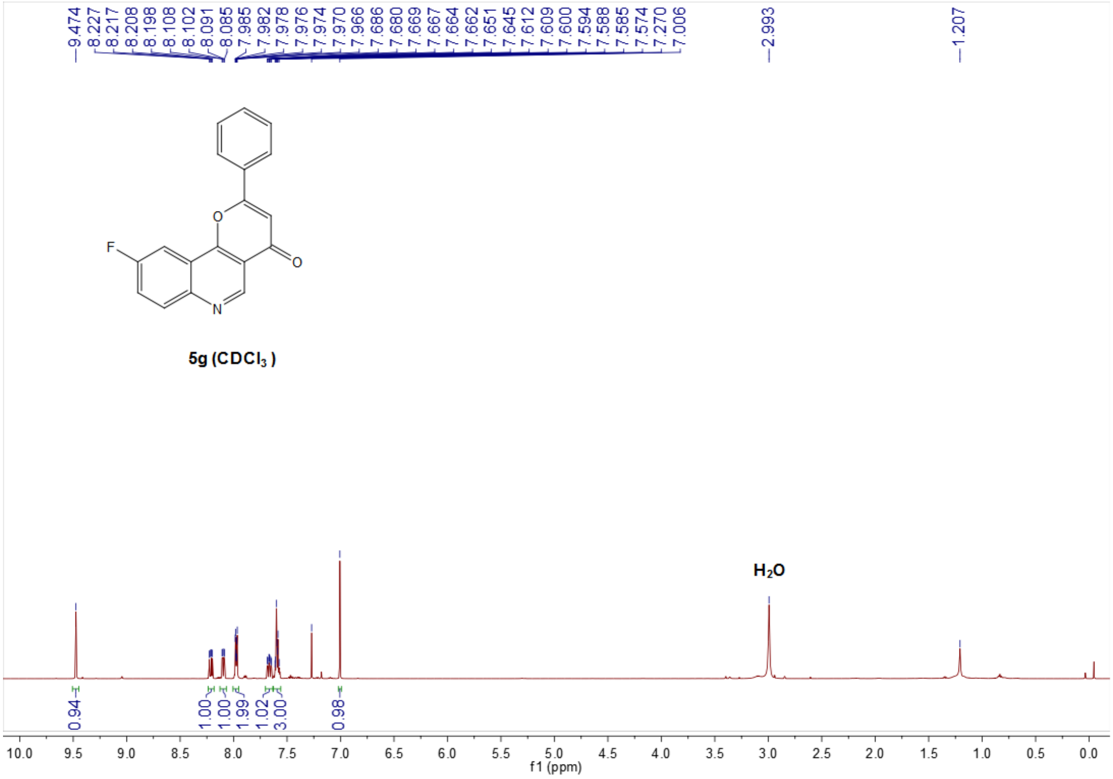
9.474
8.227
8.217
8.208
8.198
8.108
8.102
8.091
8.085
7.985
7.982
7.978
7.976
7.974
7.970
7.966
7.686
7.680
7.669
7.667
7.664
7.662
7.651
7.645
7.612
7.609
7.600
7.594
7.588
7.585
7.574
7.270
7.006
2.993
1.207
F
O
O
N
5g (CDCl3)
H2O
0.94
1.00
1.00
1.99
1.02
3.00
0.98
f1 (ppm)

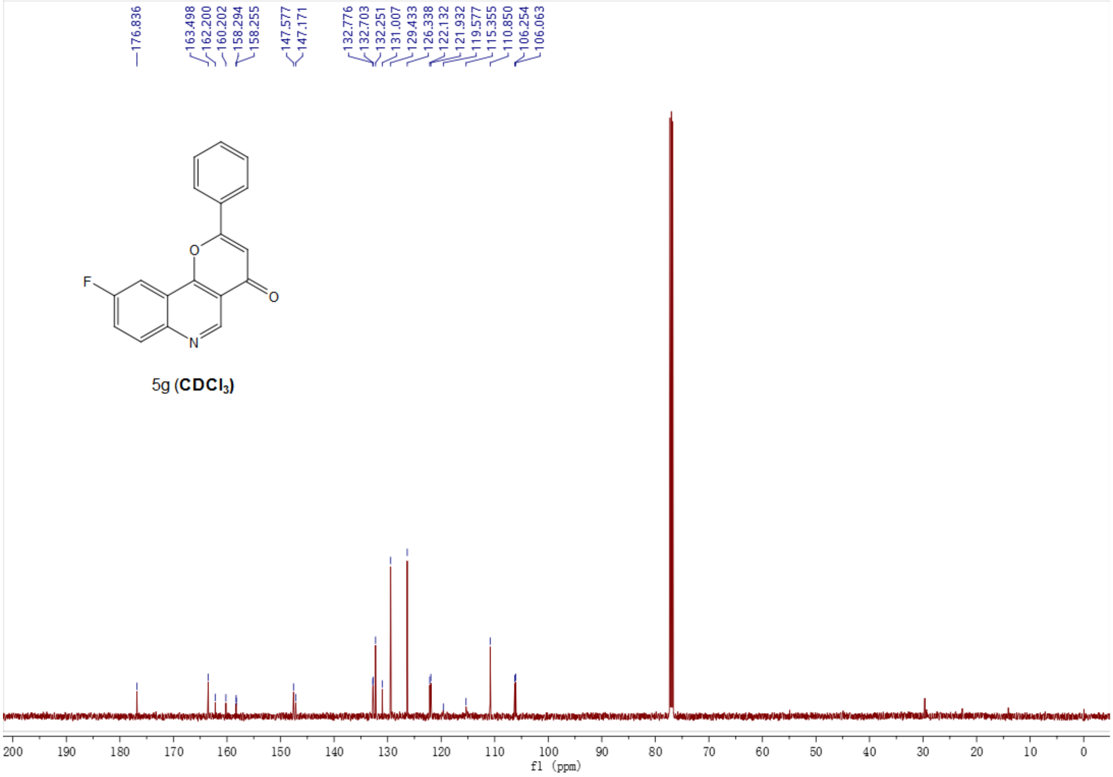
176.836
163.498
162.200
160.202
158.294
158.255
147.577
147.171
132.776
132.703
132.251
131.007
129.433
126.338
122.132
121.932
119.577
115.355
110.850
106.254
106.063
F
O
O
N
5g (CDCl3)
f1 (ppm)

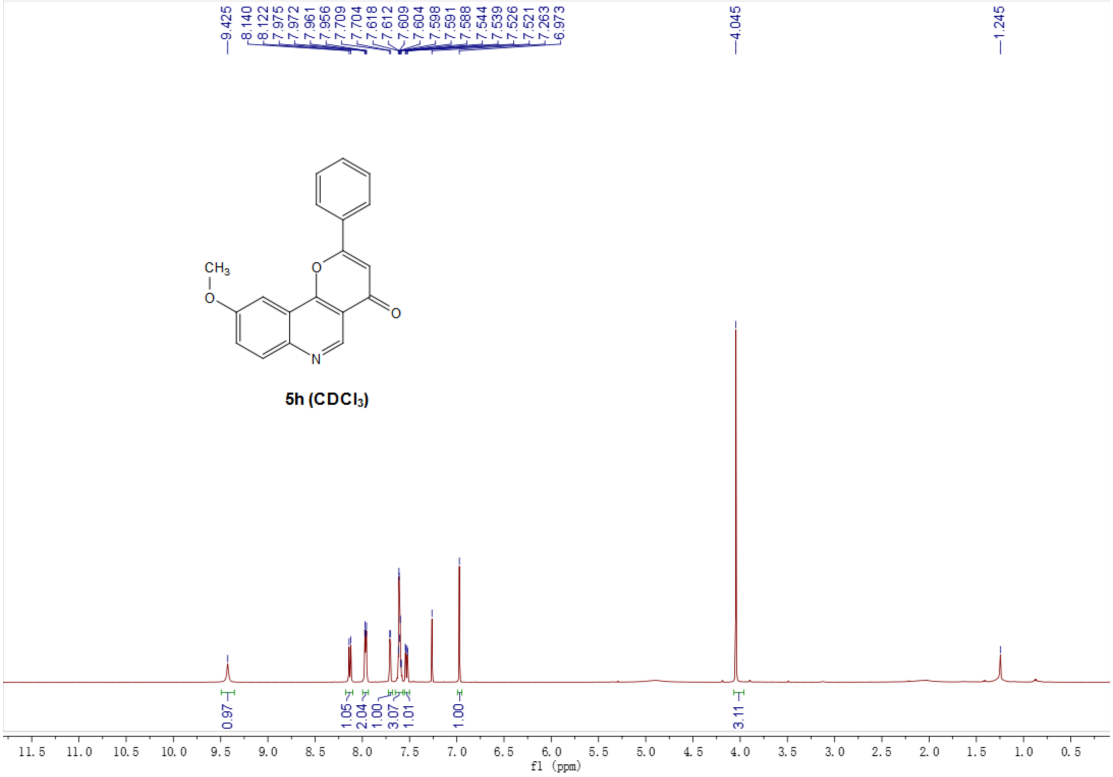
9.425
8.140
8.122
7.975
7.972
7.961
7.956
7.709
7.704
7.618
7.612
7.609
7.604
7.598
7.591
7.588
7.544
7.539
7.526
7.521
7.263
6.973
4.045
1.245
CH3
O
O
O
N
5h (CDCl3)
0.97
1.05
2.04
1.00
3.07
1.01
1.00
3.11
11.5 11.0 10.5 10.0 9.5 9.0 8.5 8.0 7.5 7.0 6.5 6.0 5.5 5.0 4.5 4.0 3.5 3.0 2.5 2.0 1.5 1.0 0.5
f1 (ppm)

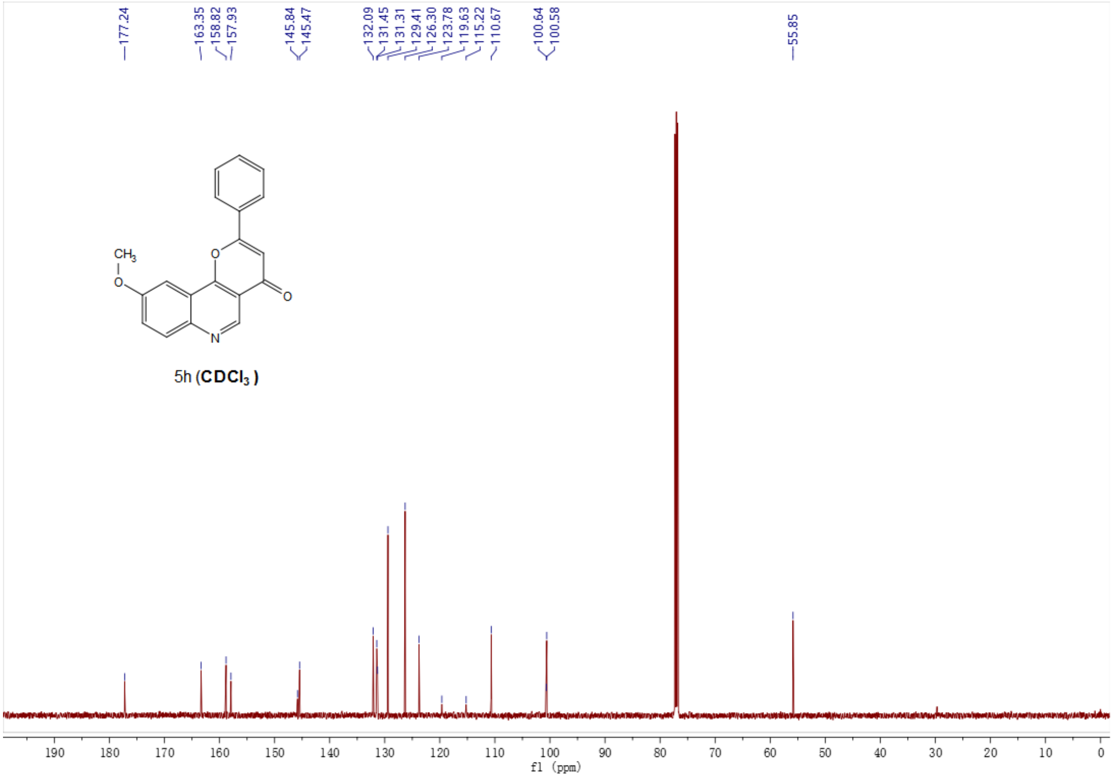
177.24
163.35
158.82
157.93
145.84
145.47
132.09
131.45
131.31
129.41
126.30
123.78
119.63
115.22
110.67
100.64
100.58
55.85
CH3
O
O
O
N
5h (CDCl3)
190 180 170 160 150 140 130 120 110 100 90 80 70 60 50 40 30 20 10 0
f1 (ppm)

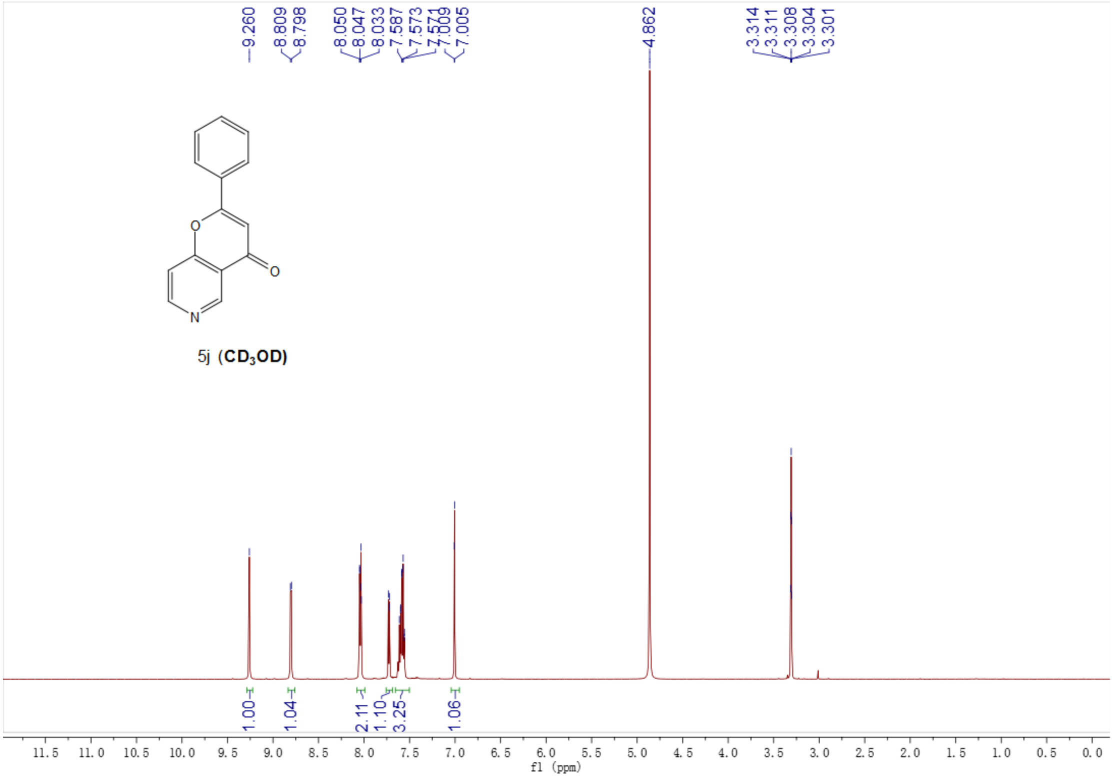
9.260
8.809
8.798
8.050
8.047
8.033
7.587
7.573
7.571
7.009
7.005
4.862
3.314
3.311
3.308
3.304
3.301
O
O
N
5j (CD3OD)
1.00
1.04
2.11
1.10
3.25
1.06
11.5 11.0 10.5 10.0 9.5 9.0 8.5 8.0 7.5 7.0 6.5 6.0 5.5 5.0 4.5 4.0 3.5 3.0 2.5 2.0 1.5 1.0 0.5 0.0
f1 (ppm)

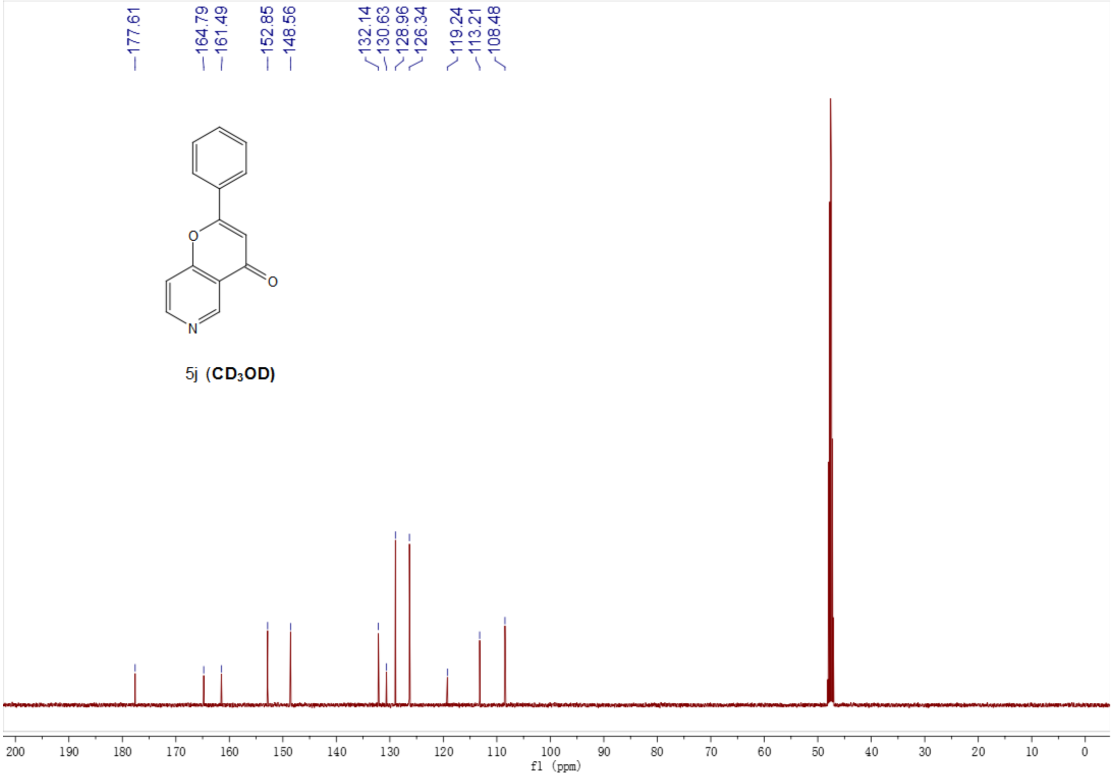
177.61
164.79
161.49
152.85
148.56
132.14
130.63
128.96
126.34
119.24
113.21
108.48
O
O
N
5j (CD3OD)
200 190 180 170 160 150 140 130 120 110 100 90 80 70 60 50 40 30 20 10 0
f1 (ppm)

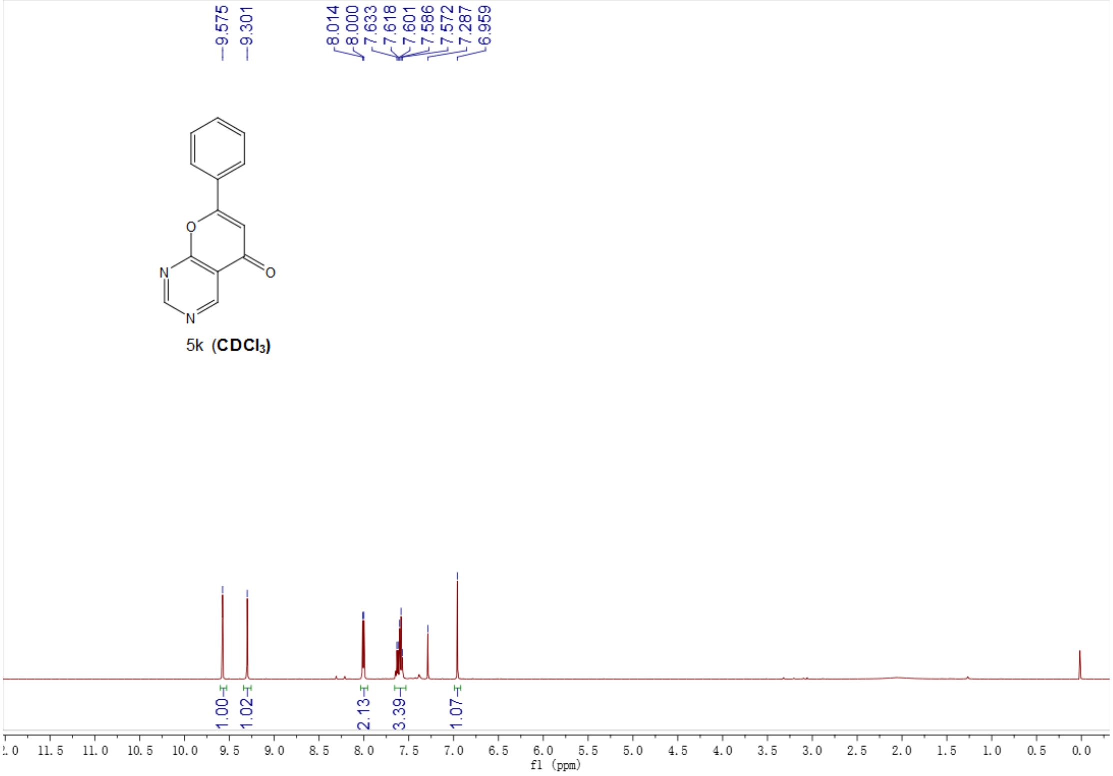
9.575
9.301
8.014
8.000
7.633
7.618
7.601
7.586
7.572
7.287
6.959
5k (CDCl3)
1.00
1.02
2.13
3.39
1.07
f1 (ppm)

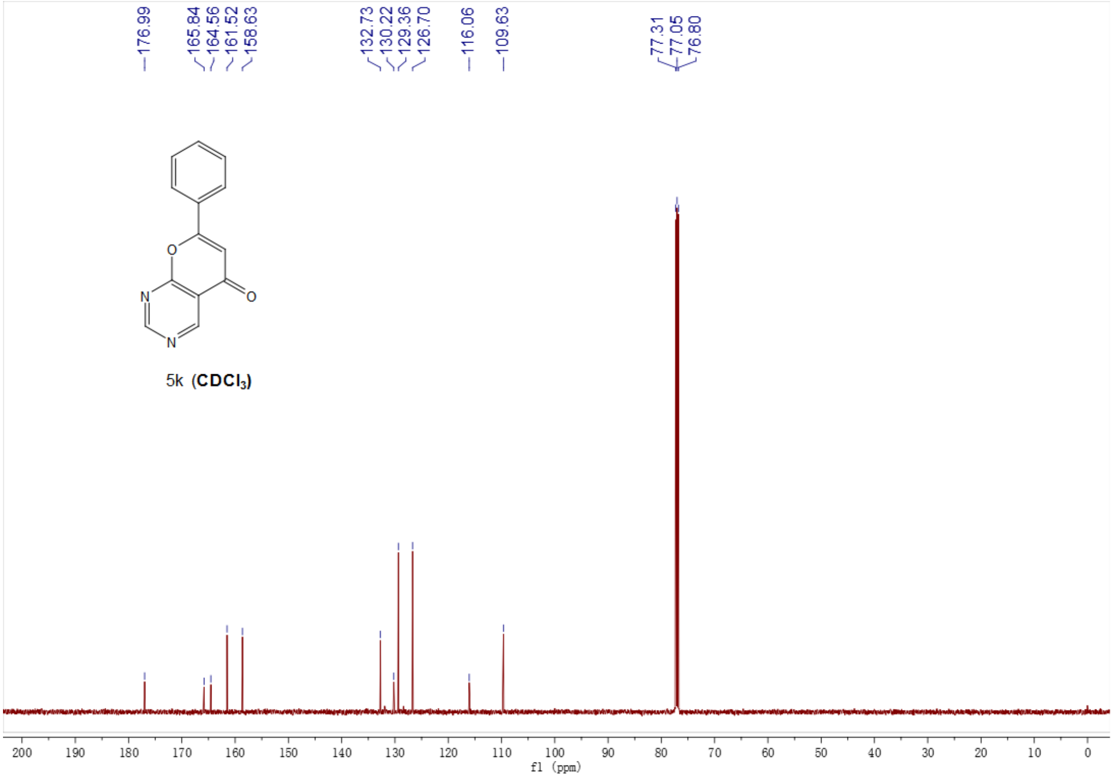
176.99
165.84
164.56
161.52
158.63
132.73
130.22
129.36
126.70
116.06
109.63
77.31
77.05
76.80
5k (CDCl3)
f1 (ppm)
